# A comparison of Affymetrix gene expression arrays

**DOI:** 10.1186/1471-2105-8-449

**Published:** 2007-11-15

**Authors:** Mark D Robinson, Terence P Speed

**Affiliations:** 1 Bioinformatics Division, Walter and Eliza Hall Institute of Medical Research, Parkville, VIC 3050, Australia; 2 Department of Medical Biology, The University of Melbourne, Parkville, VIC 3010, Australia

## Abstract

**Background:**

Affymetrix GeneChips™ are an important tool in many facets of biological research. Recently, notable design changes to the chips have been made. In this study, we use publicly available data from Affymetrix to gauge the performance of three human gene expression arrays: Human Genome U133 Plus 2.0 (U133), Human Exon 1.0 ST (HuEx) and Human Gene 1.0 ST (HuGene).

**Results:**

We studied probe-, exon- and gene-level reproducibility of technical and biological replicates from each of the 3 platforms. The U133 array has larger feature sizes so it is no surprise that probe-level variances are smaller, however the larger number of probes per gene on the HuGene array seems to produce gene-level summaries that have similar variances. The gene-level summaries of the HuEx array are less reproducible than the other two, despite having the largest average number of probes per gene. Greater than 80% of the content on the HuEx arrays is expressed at or near background. Biological variation seems to have a smaller effect on U133 data. Comparing the overlap of differentially expressed genes, we see a high overall concordance among all 3 platforms, with HuEx and HuGene having greater overlap, as expected given their design. We performed an analysis of detection rates and area under ROC curves using an experiment made up of several mixtures of 2 human tissues. Though it appears that the HuEx array has worse performance in terms of detection rates, all arrays have similar ability to separate differentially expressed and non-differentially expressed genes.

**Conclusion:**

Despite noticeable differences in the probe-level reproducibility, gene-level reproducibility and differential expression detection are quite similar across the three platforms. The HuEx array, an all-encompassing array, has the flexibility of measuring all known or predicted exonic content. However, the HuEx array induces poorer reproducibility for genes with fewer exons. The HuGene measures just the well-annotated genome content and appears to perform well. The U133 array, though not able to measure across the full length of a transcript, appears to perform as well as the newer designs on the set of genes common to all 3 platforms.

## Background

The use of Affymetrix GeneChips™ is widespread in biomedical research for profiling the expression level of thousands of genes simultaneously. The technology has been well-studied and the data processing algorithms are mature [1]. For example, Affymetrix maintains a database of nearly 10,000 (at the time of writing) scientific articles using or reviewing their technology [2], and it is arguably the single most utilized commercial DNA microarray platform.

The trend in genomic data collection has been to interrogate more and more biological features (e.g. transcripts, single nucleotide polymorphisms, proteins). The new designs from Affymetrix certainly keep to this trend, following advances in design and fabrication that allow more features on a single chip. Though one may argue that more is usually better, it is of considerable importance to ensure that the larger numbers of measurements can still provide accurate biological insights.

In this study, we compare various measures of performance of the three most recent human expression arrays: Human Genome U133 Plus 2.0 (U133), Human Exon 1.0 ST (HuEx) and Human Gene 1.0 ST (HuGene). We use two publicly available datasets from Affymetrix: an experiment consisting of 3 biological replicates each of 11 tissues and an experiment containing 3 technical replicates each of 11 RNA mixtures from brain and heart tissue [3]. Each set of RNA has been run on all three platforms. The focus of our study will be on gene-level summaries, although we acknowledge that exon arrays have applications for detecting alternative splice events, as evidenced by a number of recent publications [4,5].

### Affymetrix chip design

Affymetrix chips use 25-mer oligonucleotide probes to measure the abundance of mRNA transcripts. For the U133 and previous expression arrays, these probes occur in pairs, known as perfect match (PM) and mismatch (MM), where MM probes have a 13^th ^base that does not match the target sequence and were intended to account for non-specific binding. Under the standard annotation provided by Affymetrix, each transcript is interrogated by 11 probe pairs. Many groups prefer to use reassembled versions of the annotation where sets of probes are geared toward different databases of genes, transcripts or transcript clusters (e.g. Entrez Gene, RefSeq, Unigene) [6].

There are 3 major changes to the design for their new arrays HuEx and HuGene. First, to allow for more probes on an array, feature size has been reduced to almost one-fifth of the area (from 11 by 11 micron squares on U133 to 5 by 5 micron squares on HuEx, HuGene). We investigate the impact of this change on probe-level and gene-level reproducibility. The second significant design change is that no matching MM probes are used for every PM probe. Instead, the HuEx and HuGene arrays have allocated a small number of MM probes designed to cover the range of GC content and a number of anti-genomic probes also covering the range of GC content. Anti-genomic probes query sequence that is not present in the human genome nor in other commonly studied model organisms (mouse, rat, fruitfly, worm, Baker's yeast, Arabidopsis and *E. coli*). The absence of MM probes has implications for some summarization algorithms and also may affect background subtraction procedures. Table 1 of [1] gives a comparative list of normalization, background subtraction and summarization strategies for Affymetrix chips, and we refer to the rest of that paper for a detailed performance comparison. Our processing of the data is unaffected by the absence of MM probes (see Methods). Last of all, HuEx and HuGene probes are designed to interrogate the entire length of a gene, while probes on the U133 array are mostly at the 3' end of the gene. In the HuEx and HuGene arrays, probes are designed for each exon and can be used altogether to summarize the expression level of a gene. The labeling strategy employed by the HuEx and HuGene arrays requires sense targets to be hybridized to the chips.

**Table 1:** Mixture experiment proportions

	Mix 1	Mix 2	Mix 3	Mix 4	Mix 5a, b, c	Mix 6	Mix 7	Mix 8	Mix 9
Brain	.00	.05	.10	.25	.50	.75	.90	.95	1.00
Heart	1.00	.95	.90	.75	.50	.25	.10	.05	.00

It is worth mentioning a few more details of content contained on the HuEx and HuGene arrays. HuEx was designed with probes for virtually all confirmed or putative exonic content, with the goal of having 4 probes per *probe selection region *(PSR). Roughly speaking, a probe selection region (PSR) is a region that may be, based on all annotation available, expressed in an independent fashion. In most cases, a PSR is simply an exon. But, there are many cases where annotation (e.g. EST, gene predictions) suggests that shorter regions within an exon may need to be queried separately. In this case, a PSR is created for each region. The majority of the content on the exon array is dedicated to "speculative and predictive parts of a gene", in addition to well-known exons [7]. Though the HuEx array was not designed as simply an expression array, it will be used as one and it is important to know how well it performs as one. The HuGene array is roughly made up of the subset of the probes to the well-annotated content on the HuEx array, such as those from the curated and predicted RefSeq mRNAs. The subset of probes chosen for HuGene was based on minimizing cross-hybridization, with preference given to probes uniquely matching to the genome. More details are given later in the paper.

### Related work

Exon arrays have been compared to U133 arrays previously in at least two studies. Gardina et al. [5] highlight the differences in probe intensity distributions, suggesting that lower expressed probes see a shift upwards, which is possibly due to higher sensitivity of the new arrays. Scatter plots of gene-level summaries of matched probesets show a reasonable degree of concordance between platforms. Okoniewski et al. [8] report "high correspondence" between the HuEx and U133 arrays by comparing technical replicates of two cell lines on each platform. Using multiple methods of mapping probesets between the two platforms, they showed high correlation of fold change estimates and strong overlap of the common probesets differentially expressed between the two cell lines. By applying filters on presence calls (U133) or detection above background (HuEx), these levels of concordance are raised.

Thus, despite the major design changes, early evidence suggests that the HuEx array and U133 array give similar results, in terms of expression level and fold change estimates. To the best of our knowledge, a comparison of the HuGene array has only been considered in the Affymetrix white paper [7].

### Description of datasets

We make use of two datasets that are publicly available from Affymetrix [3]. The first dataset concerns biological replicates of 11 human tissues: brain, thyroid, breast, pancreas, prostate, heart, skeletal muscle, kidney, testis, spleen and liver. We refer to this throughout the paper as the **tissue **experiment. Three biological replicates from a commercial source were used for each tissue, giving a total of 33 samples hybridized to each array type. It is not clear whether exactly the same RNA sample from a replicate on one platform was used on the other platforms. Even if so, the samples were separately amplified and labeled. From an exploratory analysis of gene-level summaries (data not shown), no obvious correspondences between the replicates across platforms were observed, and this was not the focus of our analysis. We refer to the second dataset as the **mixture **experiment. In this dataset, commercial total RNA from brain and heart was mixed together in 9 different proportions including both sets of pure samples, as given in Table 1. The mixture at 50% brain and 50% heart was repeated 3 times. Each of the 11 mixtures (3 at 50%/50%) was split into 3 technical replicates, and amplified and labeled separately, resulting in 33 samples for each platform. There is no biological variation in the mixture replicates.

With the changes in array design, it is important to ascertain whether the HuEx and HuGene arrays have a performance similar to that of the U133 arrays. In this paper, we give a thorough comparison of the three platforms, in terms of the reproducibility of technical and biological replicates using both probe-level and gene-level summaries, and of their ability to detect changes in gene expression. The tissue dataset is used to assess biological variability. The mixture dataset is used to assess technical variability, as well as make comparisons in terms of detection of differential expression. In the absence of a spike-in experiment where the truth is known, a mixture experiment can be utilized to identify a set of truly changing genes. To assess performance, we can follow this set of genes across the range of mixture differences.

## Results

### Summary of array content

First, it is of interest to get a sense of the number of features used to interrogate mRNAs on these array types. Table 2 summarizes the number of probes and probesets represented on the 3 platforms, based on either the standard Affymetrix mapping or an Ensembl-based mapping. Affymetrix has divided annotation for the HuEx array into 3 non-overlapping groups, in decreasing order of confidence: **core **(RefSeq and full length mRNAs), **extended **(ESTs, sytenic rat and mouse mRNAs) and **full **(*ab-initio *predictions). From Table 2, we see that 80% of the HuEx array (*> *4.1 million probes) is dedicated to the lower confidence content represented by the full and extended sets of probes. In contrast, the HuGene array represents a subset of the HuEx core set only. Approximately 80% of the HuGene probes map exactly to HuEx probes. The remaining probes are unique to HuGene but measure exons already covered in the HuEx core set. Later in the paper, we make use of probesets organized according to the definition of exons and genes from Ensembl. For this, we downloaded the U133 probesets from [6] and manually created probesets for HuEx and HuGene based on genome coordinates of both the exons and probes. Details of generating Ensembl-based probesets are in the Methods section.

**Table 2:** Summary of array content

	U133	HuEx	type	HuGene
Number of Probes (Affymetrix)	604,258	1,073,146	core	844,550
	-	2,001,552	extended	-
	-	2,152,537	full	-
Number of Probesets (Affymetrix)	54,675	22,010	core	33,252
Number of Probesets (Ensembl-gene)	17,271	28,206	-	27,901
Number of Probesets (Ensembl-exon)	-	219,230	-	206,728

### Array quality

Many summaries and comparisons discussed later in the paper could be adversely affected by poorly performing chips or sets of 3 replicate chips. And, because sets of 3 replicates are reused into various pairwise comparisons, one poorly performing chip (or set of 3 chips) can affect many of the comparisons. We first assess the data quality using standard diagnostic plots [9]. Additional file 1 (pages 1–3) shows *relative log expression *and *normalized unscaled standard error plots *for each of the 3 platforms on the 33 samples in the mixture dataset.

There is the occasional chip that stands out somewhat from the rest, but in general not enough to warrant removal from the analysis. It appears from the quality assessment plots that more of the HuEx hybridizations seem to be affected. Because of this, we made a more thorough attempt to investigate the effect of the more poorly performing chips on our results. A subjective call was made on the quality of each of the 11 mixtures for each platform (see Additional file 1, page 4). Later in the paper, we highlight possible effects that these questionable or bad chips may have on the analysis.

### Probe-level intensity distributions

We begin with the tissue dataset. Because the platforms have possibly different proportions of highly expressing content (e.g. due to different numbers of probes per gene), the 33 tissue samples for each platform are background adjusted and normalized separately. We have used an robust multi-chip average (RMA) background subtraction and then quantile normalization. Full data processing details are given in the Methods section.

**Figure 1:**
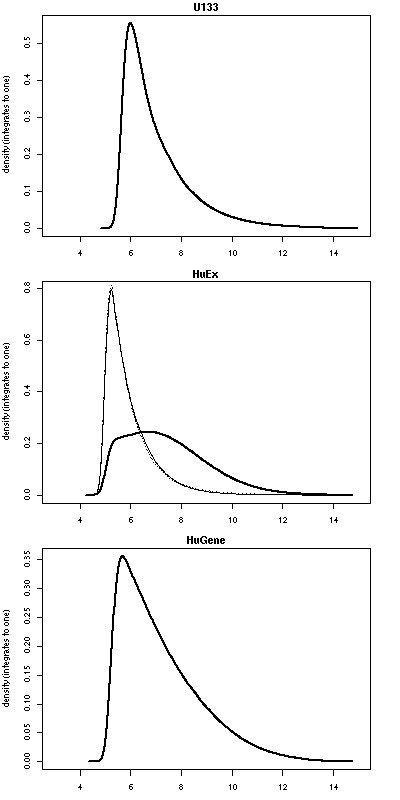
**Probe intensity distributions**. Probe-level intensity distribution of a single normalized (RMA-background corrected and quantile normalized) sample for each of the 3 platforms. For the HuEx array, the distribution are split into the core (solid line), extended (dashed line) and full (dotted line) probes. The extended and full probe intensity distributions are practically indistinguishable.

Figure 1 presents the probe-level PM intensity distributions for a single normalized sample from each of the 3 platforms. The intensity distributions for the HuEx array have been separated into probes corresponding to core, extended and full probesets. One can see that the probes from the majority of the full and extended probesets (a total of 80% of the probes on the array) have intensity close to background, which is in line with our expectations. For our purposes, it is not important that the intensity distributions across platforms have slightly different central values since we do not make direct comparisons at this level. However, it is interesting to note the distributions have slightly different shapes. Specifically, a larger proportion of probes enter into the high end of the intensity scale for the HuEx core and HuGene arrays, suggesting that querying across the entire length of the gene has merit.

### Reproducibility of technical and biological replicates at different levels of summarization

**Figure 2:**
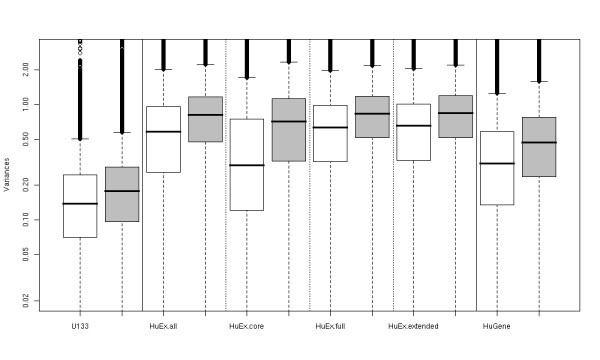
**Probe-level reproducibility**. Boxplots of the distribution of pooled variances across probes for each of the 3 platforms for the technical replicates of the mixture experiments (white) and the biological replicates of the tissues experiment (grey). The HuEx probes are split into core, extended and full.

It is of interest to compare the reproducibility of probe-, exon- and gene-level summaries across platforms. One way to do this is to calculate variances for each probe, with lower variances suggesting greater reproducibility. We calculate the mean intensity for each set of 3 replicates, and the 11 variances, each on 2 degrees of freedom (since there are 11 tissues and 11 mixtures), thus accounting for different intensity levels across tissues/mixtures. These residual variances are then averaged over the 11 estimates to arrive at a single variance for each probe, for each platform, having 22 degrees of freedom. Figure 2 summarizes the distribution of pooled probe-level variances for both experiments across the 3 platforms, with the HuEx probes split into the 3 levels of annotation. We have examined the homogeneity of the variances for each probe across mixture/tissues proportions, and only a very small proportion are non-homogeneous (data not shown).

There are a number of interesting observations that can be made on the basis of these displays. We know that the U133 feature sizes are nearly 5 (11^2^/5^2^) times larger than those on the other platforms. According to the medians of the probe-level variance distributions for the technical replicates, the U133 array has 2.1 and 2.2 times lower variance than the HuEx core probes and HuGene probes, respectively. HuEx's extended and full probes have higher variances, as would be expected, since a larger proportion of them are near background. A very similar mean-variance relationship is observed across platforms (see Additional file 1, page 5), though shifted in the variance, as observed in the boxplots of Figure 2. Since the tissue experiment uses biological replicates and the mixture experiment uses technical replicates, it is not surprising that the variances are lower for the mixture experiment. However, it is not clear why biological variation contributes quite a bit less to the U133 platform than to the whole-transcript-based platforms. It is possible that the new labeling strategy contributes to this, since HuEx and HuGene are similarly affected. It is also possible that the samples were processed at different times or handled differently. To assess the effect of background adjustment and normalization on the HuEx array, we processed the data for the HuEx core probes in two different ways: one including all probes, and one treating the array as if it were made up of core probes only. This had very little effect on the intensity or variance distributions (data not shown), so the fact that the HuEx array contains 80% of probes near background is inconsequential to probe level summaries.

**Figure 3:**
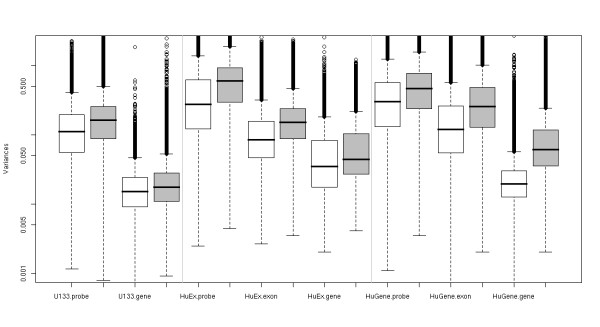
**Probeset-level reproducibility**. Boxplots of the distribution of pooled variances for probe-, exon- (HuEx and HuGene only) and gene-level summaries for each of the 3 platforms for the technical replicates of the mixture experiments (white) and the biological replicates of the tissues experiment (grey). Probesets are based on Ensembl genes.

Next, we compare variances at different summarization levels. The RMA model can work with probesets that are exons or probesets that encompass all the probes for a given gene. Gene-level summaries should be less variable than exon-level summaries, which in turn should be less variable than probe-level intensities. As before, we average variances across the 11 sets of replicates for either the exon-level or the gene-level summaries, for both experiments. Using custom created probesets based on Ensembl annotation (see Methods), we can compare exon-level summaries (for the HuEx and HuGene arrays) as well as gene-level summaries. The numbers of probes per gene or exon and the median probe- and gene-level variances for technical replicates are summarized in Table 3. Figure 3 illustrates the variances at different summarization levels across platforms for both experiments using Ensembl-based probesets. Each boxplot shows the variance distribution (on the log scale) over all probes or (Ensembl-based) probesets for the corresponding platform, according to Table 3. Interestingly, constitutive exons (see Methods) have a variance distribution that is effectively identical to that of the non-constitutive exons (data not shown). At both the probe- and gene-level, the U133 array has the best reproducibility. Due to the larger number of probes per gene, the gene-level reproducibility on the HuGene array is effectively the same as U133. It appears that more probes per gene on the HuEx array is not effective in reducing the variance to the desired extent. The median probe-level variances are essentially the same on the HuEx (core) and HuGene platforms, however, the gene-level median variance is 1.8 times higher on the HuEx array compared to HuGene, despite having more probes per gene. It appears that is this largely due to the distribution of number of probes per gene for Ensembl-based probesets. Generally, more probes for a gene leads to greater reproducibility (see Additional file 1, page 6). However, the HuEx and HuGene platforms differ drastically in the distribution of number of probes per gene (see Additional file 1, page 7). The HuEx array is designed for exon coverage, so longer genes always have more probes. The HuGene array is designed to have most probesets with the average number of probes per gene (~26), regardless of the length of the gene. In fact, there are several thousand "single-exon" genes (according to Ensembl) that have only 4 probes on the HuEx platform but more than 10 on the HuGene platform (see Additional file 1, pages 8–9). These differences in the probe selection for the HuEx and HuGene arrays largely account for the shift in gene-level variances.

**Table 3:** Summary of probe- and gene-level variances for technical replicates

	U133	HuEx	HuGene
Average Number of Probes per Gene	16.1	44.8	26.8
Average Number of Probes per Exon	-	5.8	3.7
Median Probe-Level Variance	0.139	0.306	0.308
Median Exon-Level Variance	-	0.086	0.119
Median Gene-Level Variance	0.015	0.035	0.020

Figure 3 also highlights the possibility of using the HuGene array as an exon array for well-annotated content. The HuGene platform has probes designed across the full length of a transcript. The median exon-level variance is only 1.4 times larger, despite the smaller number of probes. In addition, HuGene probes cover ~95% of the exons covered in full HuEx array, according to Ensembl annotation.

From Figure 3, it again appears that the contribution of biological variability in the tissue experiment represents different proportions of the total variability at the gene-level across the platforms. The U133 array appears to propogate a smaller amount of biological variability, whereas there is a larger difference between technical and biological variances from the HuEx and HuGene arrays. It is possible that some artifact in the sample handling or biological samples has introduced a higher level of noise, or it could be that there is truly more biological variability that the newer platforms are now measuring.

### Finding differentially expressed genes

For the rest of the paper, we focus on the mixture dataset. To allow for an easier matching of probesets between platforms, we employ the Ensembl gene-centric probesets discussed in the previous section. We restrict our comparison to the same 16,941 Ensembl genes (the intersection of the 3 sets of Ensembl identifiers) measured by all three platforms. This number is slightly larger than the 15,585 matched probesets used in the Affymetrix white paper [7]. A scatter plot of the averaged gene-level summaries for one particular mixture is given in Additional file 1 (page 10), highlighting the concordance between platforms. We observe a correlation of 0.80 between U133 and either of HuEx and HuGene despite a somewhat non-linear relationship. The non-linearity in gene-level summaries is partly due to observing genes with slightly higher intensities on HuEx and HuGene arrays, as noted previously by [5], suggesting a higher sensitivity for the new platforms. The correlation between HuEx and HuGene is high at 0.98, as expected given they are designed similarly and in fact they share a large number of probes.

**Figure 4:**
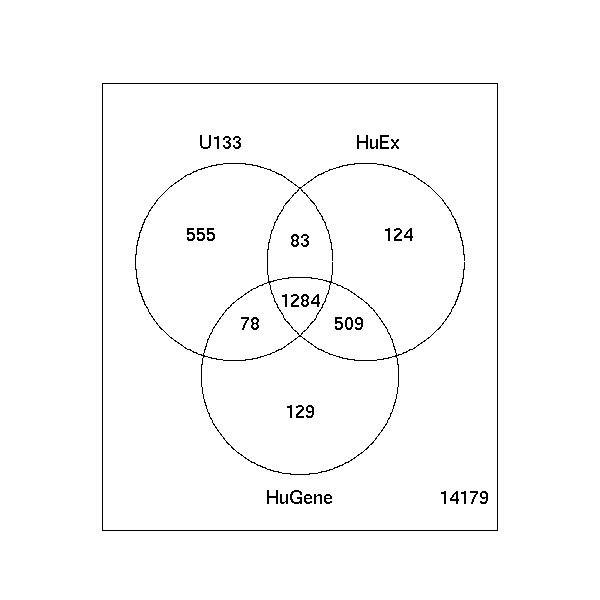
**Overlap of top 2,000 differentially expressed genes**. Venn Diagram showing the overlap of top moderated *t*-statistics between pure brain and pure heart samples using Ensembl mappings.

Next, we consider the task of finding differentially expressed (DE) genes between the brain and heart samples using the mixture experiment. Using the common set of Ensembl genes, we computed moderated *t*-statistics using *limma *[10] for the changes between the 3 pure brain (Mix 9) and 3 pure heart (Mix 1) samples. Figure 4 shows the number of genes that overlap, based on the top 2,000 moderated *t*-statistics from each platform. Because of the differences in array design, it is inevitable that the U133 chips will measure slightly different effects, whereas the HuEx and HuGene should generally measure the same transcripts. Despite this, the intersection of all 3 array types contains nearly 65% of each platform's DE genes. U133 has 25% uniquely represented DE genes whereas the DE genes from the HuEx and HuGene arrays overlap by ~80%, as expected. In comparing the top ranked 1,000 and 3,000 moderated *t*-statistics for each platform, the percent overlap results are very similar (data not shown).

In the next section, we follow a set of DE genes across all the mixtures as a means to assess performance. Before doing this, it is interesting to compare the number of genes deemed differentially expressed (DE), given that by restricting to the same set of genes, each platform will have the same penalty for multiple testing. We used the moderated *t*-statistics from *limma *[10] to compute raw *p*-values, which are then adjusted by a Benjamini-Hochberg correction. Table 4 summarizes the number of genes at different estimated false discovery rates. Here, we see first of all that there are a large number of DE genes (~50% of the common set), giving a large pool for us to assess performance with and that, by using the same false discovery rate (FDR) cutoff, HuGene consistently calls the largest number of genes and HuEx calls the smallest. The reason for the difference is due in part to the inner workings of *limma*. Specifically, a higher estimated prior degrees of freedom, which results when the variances are more uniform (i.e. genes have approximately the same variance), can lead to more genes called as DE, since the threshold on the absolute *t*-statistics at a given FDR, everything else equal, is lower. For the pure brain-pure heart comparison (Mix 1-Mix 9), the residual variances on the HuGene platform are most uniform, allowing more genes to be called DE at a preset FDR threshold. The higher uniformity of variances for the HuGene platform is presumably due to the very uniform number of probes per gene. To test whether HuGene data consistently had more uniform variances, we compared the estimated prior degrees of freedom across the set of 36 possible pairwise mixture comparisons (not including Mix 1 and Mix 9). The estimated prior degrees of freedom was consistently higher for comparisons on the HuGene array, as shown in Additional file 1 (page 11). If gene-level variances are consistently more uniform in many HuGene datasets, that could prove advantageous for the platform in many applications.

**Table 4:** Numbers of differentially expressed genes

	U133	HuEx	HuGene
FDR = .05	10,991	10,183	11,252
FDR = .01	9,359	8,474	9,764
FDR = .001	7,416	6,694	8,181

### Detection rates

For our analysis of detection rates, we fix the set of DE genes according to the comparison between pure brain and heart samples and follow these genes across several other comparisons. In order to not bias towards a particular platform, we select 3 sets of DE genes, one for each platform separately and follow each set across all the RNA mixtures for the corresponding platform. To make the comparisons fair, we make pairwise comparisons between all sets of 3 samples (after setting aside the pure brain and pure heart sets). Each comparison is thus made up of total of 6 samples: 3 technical replicates of one mixture condition against 3 technical replicates of another mixture condition. Since there are 9 mixture levels (Mix 2 to Mix 8, with Mix 5 repeated 3 times), we have 36 possible pairwise comparisons, independent of the 2 sets of pure mixtures (Mix 1 and Mix 9). Our strategy is similar to the approach used by Affymetrix in their white paper [7], except that they selected genes using all mixtures. We have selected our set of "true" DE genes independently of the dataset used to evaluate performance.

For our purposes, detection is defined as the ability to call a truly DE gene, here predefined independently, as DE for a given comparison. We compare the ability to detect gene expression changes by following our sets of truly DE genes across mixtures where the degree of difference can be small.

**Figure 5:**
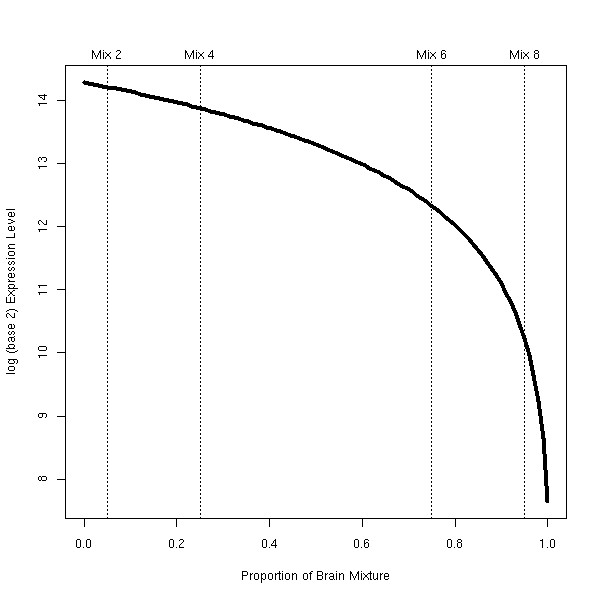
**Illustration of asymmetry for a hypothetical gene**. A hypothetical gene that is differentially expressed between heart and brain (highly expressed in heart). Y-axis gives the expression level on the log-scale. X-axis is the proportion of brain RNA making up the mixture (heart RNA makes up the remainder). Highlighted with the dotted lines are the mixtures with 5%, 25%, 75% and 95%, showing that finding differences between 5% and 25% (a 20% difference) and 75% and 95% (also a 20% difference) have very different degrees of difficulty.

**Figure 6:**
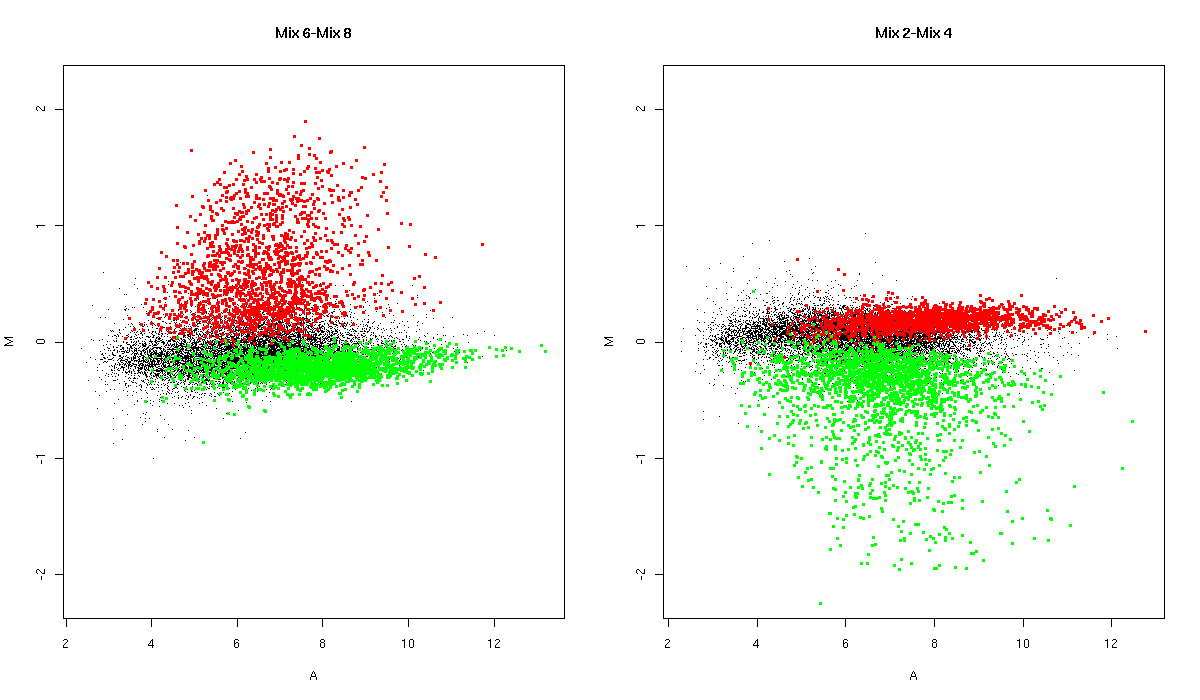
**MA plots for different 20% mixtures**. M (log-fold change) versus A (intensity) for two 20% mixture difference comparisons: Mix 6-Mix 8 and Mix 2-Mix 4. Shown here is the HuEx data only. Genes expressed significantly higher in the pure heart samples (2,218) are shown in red and brain-expressed genes (1,782) are shown in green. In terms of the difficulty in detecting differential expression, the two 20% mixtures are very different.

An asymmetry is introduced when considering expression summaries on the log scale. We have found it important to summarize the results in terms of the difficulty of the problem. Figure 5 illustrates a hypothetical truly DE gene that is highly expressed in heart tissue and lowly expressed in brain tissue and, assuming linearity, the noiseless profile that should be observed in our processing of the data (log-scale). Shown on the plot are the mixtures at 5% (Mix 2) and 25% (Mix 4) brain and at 75% (Mix 6) and 95% (Mix 8) brain. Making comparisons of the Mix 2 and Mix 4 samples (a 20% difference) and the Mix 6 and Mix 8 samples (also a 20% difference) are very different for this particular gene, in terms of the difficulty of finding differential expression. While it may be easy to detect this difference in Mix 6-Mix 8 comparison (here, a difference of *>*2 on the log-scale), it may be very difficult to detect this difference in the Mix 2-Mix 4 comparison (here, a difference of *<*0.5). So, we split the 36 possible comparisons into easy and hard, based on two sets of genes (i.e. whether the curve ascends or descends) and whether it is the first half or second half of the curve (from Figure 5). Some easy comparisons are not considered since they are so large that they do not distinguish the platforms. Figure 6 shows MA plots to highlight the asymmetry in another way for the Mix 2-Mix 4 comparison and the Mix 6-Mix 8 comparison, both 20% differences, with sets of *brain *and *heart *genes highlighted. A discussion of how these genes were selected is given below. It is quite evident that the difficulty of finding differential expression between two mixture samples of the same difference (here, 20%) is dependent on the direction of the change and the initial expression level. Table 5 gives the comparisons that we are considering and how they are stratified into easy and hard. Note that the difficulty in detecting expression changes is not solved by using the linear scale. The reason for using the log scale is to make the assumption of common variance (as used in *limma*, for example) more realistic. Typically, variances increase with intensity and detecting a 20% difference at high expression values would remain difficult on the linear scale.

**Table 5:** Difficulty of pairwise mixture comparisons

	MD	Heart Genes	MD	Brain Genes
Easy Comparisons	.05	Mix 7-Mix 8	.05	Mix 2-Mix 3
	.15	Mix 6-Mix 7	.15	Mix 3-Mix 4
	.20	Mix 6-Mix 8	.20	Mix 2-Mix 4
	.25	Mix 5-Mix 6	.25	Mix 4-Mix 5
	.40	Mix 5-Mix 7	.40	Mix 3-Mix 5
	.45	Mix 5-Mix 8	.45	Mix 2-Mix 5
Hard Comparisons	.05	Mix 2-Mix 3	.05	Mix 7-Mix 8
	.15	Mix 3-Mix 4	.15	Mix 6-Mix 7
	.20	Mix 2-Mix 4	.20	Mix 6-Mix 8
	.25	Mix 4-Mix 5	.25	Mix 5-Mix 6
	.40	Mix 3-Mix 5	.40	Mix 5-Mix 7
	.45	Mix 2-Mix 5	.45	Mix 5-Mix 8
Comparisons Not Considered	.50	Mix 4-Mix 6	same as heart genes	
	.65	Mix 3-Mix 6		
	.65	Mix 4-Mix 7		
	.70	Mix 2-Mix 6		
	.70	Mix 4-Mix 8		
	.80	Mix 3-Mix 7		
	.85	Mix 2-Mix 7		
	.85	Mix 3-Mix 8		
	.90	Mix 2-Mix 8		

In our first analysis, we compare *detection rates *across platforms. Since each platform has a large number of total DE genes, we followed the top 4,000 genes (according to adjusted *p*-value) across the pairwise comparisons of interest. Detection rate for a given mixture comparison is defined as the proportion of the initial set of 4,000 **true **DE genes that are below a preset cutoff. Therefore, detection rate is another term for recall or sensitivity. In our case, one might call it apparent sensitivity, since we use data to determine our set of true DE genes to start with.

**Figure 7:**
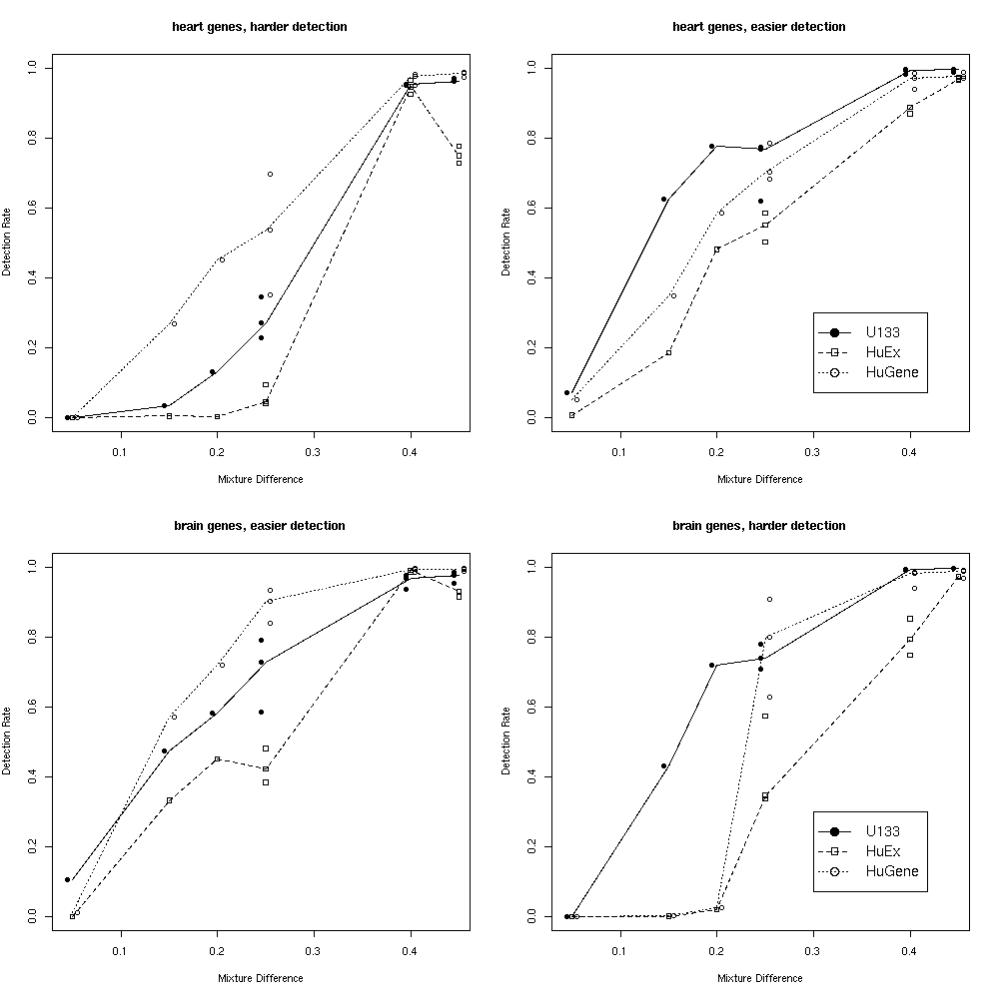
**Detection rates**. Detection Rates (proportion of previously selected "true" genes for each platform that are below the set FDR cutoff) stratified by brain and heart and "easy" and "hard" detection problems. X-axis is the mixture difference of the comparison. Y-axis gives the detection rate.

In this analysis, we used a 5% FDR threshold for all comparisons, based on the adjusted *p*-values. We have only considered comparisons where the mixture difference is smaller than 50% since the larger differences do not separate the platforms. Figure 7 shows detection rates as a function of the difference in mixtures for the top 4,000 genes, split by brain and heart genes, and into easy and hard detection problems for these genes. In general, as the degree to which two mixtures are different increases, more of the true DE genes are detected as changed. Of the 4000 genes for each platform, 1,885, 1,782 and 1,936 genes, respectively for the U133, HuEx and HuGene arrays are designated as "brain" genes. The sets of genes we consider here are different for each platform, though they largely overlap, as noticed previously. Though it is not particularly evident from Figure 7 that the "easy" detection problems always give higher detection rates, it is quite clear when you consider fewer top genes. Detection rates for the top 1,000 and 2,000 genes are shown in Additional file 1 (pages 12–13). The major trends are conserved with an increase in the overall detection rates. Also in the Supplementary materials, we made an attempt to highlight comparisons that may contain one or more chips of lower quality. It appears that some but not all of comparisons with unexpectedly low detection rates observed can be attributed to array quality.

In terms of detection rates, U133 and HuGene have the best overall performance, where each are top-ranked in 2 of 4 comparisons and second-ranked in the other 2 comparisons. With smaller sets of genes, U133 appears to be marginally better.

### ROC analysis

Next, we consider a very similar analysis to detection rates except we form a "matching" set of deemed non-DE genes. Under this framework, we can generate a receiver operating characteristic (ROC) curve for each comparison based on separating the DE (positives) and non-DE (negatives) genes, thereby not relying on a particular cutoff. We start with the same 4,000 truly DE genes from the previous section. Typically, the DE genes are biased towards higher expression, so we pick a set of non-DE genes that are matched in intensity (here, intensity is defined as the average log-expression over the 6 pure samples). From the comparisons of pure samples (Mix 1-Mix 9), we ranked genes according to adjusted p-values. The top 4,000 make up the true DE set, as before. From the lowest 8,000 genes (i.e. genes designated as non-DE), we devised an approximate weighted sampling scheme (with weights based on the DE and non-DE intensity distributions) to select the 4,000 non-DE genes. Details of the weighted sampling are given in the Methods section. We then follow the set of 8,000 genes (4,000 DE, 4,000 non-DE), chosen separately for each platform, across the comparisons of interest.

We consider the problem of separating the positives and negatives, preset from the Mix 1-Mix 9 comparison, across each pairwise mixture comparison of interest. This ultimately leads to an ROC curve for each comparison. Since we have several comparisons, we wish to summarize each ROC curve with a single value. One common way to summarize ROC curves is the area under the curve (AUC). In practice, we are typically not tolerant of false positive (FP) rates beyond 10%, so we have summarized each ROC curve with a partial area, the area up to a FP rate of 10% and scale each area to have a value between 0 and 1 [11].

**Figure 8:**
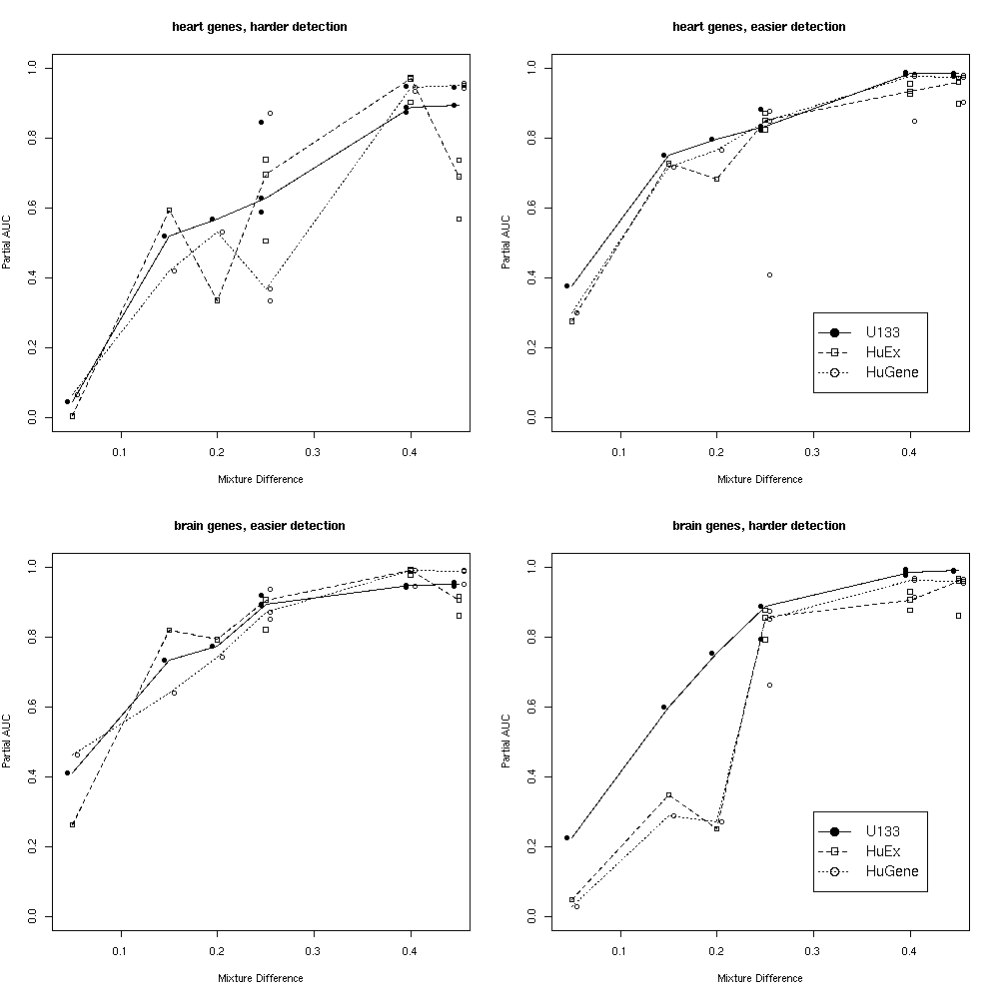
**ROC analysis**. Partial AUCs (area under the ROC curve up to FP = 10%, normalized to have a maximum possible value of 1) stratified by brain and heart and "easy" and "hard" detection problems. X-axis is the mixture difference of the comparison. Y-axis gives the pAUC.

Figure 8 gives partial areas (pAUCs) for the same stratification of genes (heart/brain) and comparisons (easy/hard) as in the previous section (Table 5). Overall, these comparisons show that, again, as the degree to which two mixtures are different increases, the truly DE genes and the non-DE genes are more easily separated. Additionally, these comparisons reveal that the platforms perform more similarly than suggested by detection rates. This is partly due to the different behaviour of the non-DE genes for some comparisons (see Additional file 1, page 14). Despite these differences, it appears that the platforms have very similar performance in terms of separating pre-defined sets of DE and non-DE genes, with U133 having a slight advantage. pAUCs for the top 1,000 and 2,000 DE genes (with corresponding matched sets of non-DE genes) are given in Additional file 1 (pages 15–16), again showing very similar results.

## Conclusion and Discussion

We made a thorough performance comparison of the three most recent human gene expression chips available from Affymetrix: Human Genome U133 Plus 2.0, Human Exon 1.0 ST and Human Gene 1.0 ST. First, we have compared reproducibility at the probe-, exon- (where possible) and gene-level using publicly available data on technical and biological replicates. We then focussed on gene-level performance, which included the overlap of DE genes from a set of RNA samples that were run on all 3 platforms.

Summarization (gene- or exon-level) relies on pre-defined probesets. The question of how and what probes are organized into a probeset is a complex one. One previously proposed strategy for the HuEx array removed probes entirely, employing a hierarchical clustering approach to select only those probes that agree to some extent with the rest [12]. The robust fit of the RMA model will account for this smoothly by downweighting probes that disagree with the majority. Another strategy, in light of the probe- to gene-level improvement of the HuGene array, and the fact that the HuGene probes are largely a subset of the HuEx probes, is to use that subset of probes where possible for the HuEx array. However, this is not always possible since the HuGene has a collection of probes unique to the platform. Another approach is to use constitutive exons only in gene-level summaries, but it does not appear that these are measured with any higher precision. Many of the exons from our Ensembl mapping include 2 or more probe selection regions from Affymetrix. These effects are important and are worthy of further study, but are beyond the scope of our study, as we aimed to make general comparisons based on standard processing.

Probe-level reproducibility, both on technical and biological replicates, is better on the U133 array doubtless due to the larger feature size. Based on probesets organized to target the Ensembl gene collection, the HuGene array makes effective use of its ~27 probes per gene to give gene-level reproducibility quite close to that of the U133 array. There are major differences in the distribution of number of probes per gene between the HuEx and HuGene arrays, which contributes to the different levels of reproducibility at the gene level. Because the HuEx is designed for exon coverage, the number of probes per gene increases with the number probe selection regions for that gene. In the HuGene array, the number of probes is designed to be more uniform, regardless of the number of exons. As a result, there are a few thousand single-exon genes (in the Ensembl gene catalogue) that are represented by a near-average number of probes on the HuGene array, but only 4 probes on the HuEx array, which affects the distribution of gene-level variances.

Like HuEx, HuGene is designed to measure expression over the full length of a transcript. Consequently, there is the possibility that HuGene array could itself be used as an economical exon array for just the well-annotated content. Its exon-level reproducibility is not much worse than the full HuEx array and the coverage of human exons is high. Clearly, further study would be required to determine whether HuGene could detect alternative splice events reliably. The main advantage of the HuEx array is its ability to measure practically every known or predicted exonic sequence in the genome.

It appears that the additional variance of biological replicates over technical replicates is smaller on the U133 platform. This may be due to the different labeling strategy on the HuEx and HuGene arrays, but a more directed experiment would be required to verify this. Another explanation is that HuEx and HuGene are more sensitive and simply uncover more true biological variability.

In overlapping DE genes across platforms for the same dataset, a high concordance is observed, as noted previously [8]. Because of the similar design, the DE genes overlap at a higher rate between the HuEx and HuGene arrays, than either with the U133 array, even when using Ensembl-centric summaries. For a given FDR using *limma*, the HuGene array calls the most DE genes between the pure heart and pure brain samples. This is due largely to the fact that estimated residual variances are more similar and more sharing between genes is done by the empirical Bayes model.

Lastly, we compared platforms in terms of detecting expression changes and the ability to separate pre-defined sets of truly changing and non-changing genes. In presenting our results, we highlighted the asymmetry introduced by adopting the log scale. While it appears that U133 and HuGene show some slight advantage based on detection rates, the pAUC performances of all 3 platforms are very similar. In summary, the extra probes on the HuEx and HuGene array seem to regain most of the performance lost from having smaller individual features. Processing the HuEx data into gene-level summaries may benefit from judicious probe selection. All platforms have similar performance in distinguishing between DE and non-DE genes according to a mixture experiment.

## Methods

### Datasets

The tissue and mixture datasets are available from [3]. Some additional descriptions of the experiments can be found from the Affymetrix website as well as within the white paper [7].

### Data processing

For all the gene- or exon-level summaries, we process the data using the linear model from RMA, but fit robustly using probe level models [13] instead of median polish. We use the R package aroma.affymetrix [14], which allows essentially unlimited numbers of large Affymetrix datasets to be analyzed using persistent memory.

Probe level models are fit to RMA-background corrected and quantile normalized data to get gene-level (or exon-level) summaries. Affymetrix makes unsupported CDF files available for U133 and HuGene chips. The initial CDF file for HuEx was created from the 'mps' annotation that can be downloaded from the Affymetrix downloads page [15].

### Creation of ensembl-centric probesets

Version 9 of the Ensembl-centric CDF files were used for U133 [16]. Using Biomart [17], tables of gene and exon identifiers with corresponding genome coordinates were downloaded to a file. Genome coordinates for each of the HuEx and HuGene probes are given in the ".probe.tab" files available from Affymetrix. To associate probes with exons, we compared genome coordinates, requiring a probe to be fully within an exon to be kept.

The gene to exon to probe CDFs were created in a nested way such that they can be used within 'ExonRmaPlm' objects in the aroma.affymetrix package. See [14] for more details. This allowed us to easily create exon-level summaries using the mergeGroups = F tag or gene-level summaries using the mergeGroups = T tag.

The custom made Ensembl-based CDFs can be downloaded from [18].

### Weighted sampling

In order to match the set of non-DE genes in intensity to the DE genes, we adopted an approximate weighted sampling procedure. First, smoothed densities of the intensities of the 4,000 DE genes and the 8,000 non-DE genes were calculated for the same grid of points using the density function in R. For each gene in the non-DE set, a weight was given according to the ratio of densities (DE to non-DE) as a function of intensity. 4,000 genes were selected from the 8,000 according to these weights, giving a sample that has approximately the same intensity distribution.

### Constitutive exons

When downloading the full list of human exons from BioMart, a column was selected to indicate whether an exon was considered constitutive or not. According to the BioMart documentation, this is determined simply based on whether or not an exon is present in all transcripts for a given gene. Approximately 52% of the exons in the human database are constitutive.

### Data analysis

All data analysis was performed in R [19]. The quality assessment plots (see Additional file 1, pages 1–3) are created using functions from the affyPLM package. The plots in Additional file 1, page 5, are created using the smoothScatter function of the geneplotter R package. The pooled variances are calculated as:

PVg=∑k=1K(nk−1)sgk2∑k=1K(nk−1)=1K∑k=1Ksgk2
 MathType@MTEF@5@5@+=feaafiart1ev1aaatCvAUfKttLearuWrP9MDH5MBPbIqV92AaeXatLxBI9gBaebbnrfifHhDYfgasaacPC6xNi=xI8qiVKYPFjYdHaVhbbf9v8qqaqFr0xc9vqFj0dXdbba91qpepeI8k8fiI+fsY=rqGqVepae9pg0db9vqaiVgFr0xfr=xfr=xc9adbaqaaeGacaGaaiaabeqaaeqabiWaaaGcbaGaemiuaaLaemOvay1aaSbaaSqaaiabdEgaNbqabaGccqGH9aqpjuaGdaWcaaqaamaaqadabaGaeiikaGIaemOBa42aaSbaaeaacqWGRbWAaeqaaiabgkHiTiabigdaXiabcMcaPiabdohaZnaaDaaabaGaem4zaCMaem4AaSgabaGaeGOmaidaaaqaaiabdUgaRjabg2da9iabigdaXaqaaiabdUealbGaeyyeIuoaaeaadaaeWaqaaiabcIcaOiabd6gaUnaaBaaabaGaem4AaSgabeaacqGHsislcqaIXaqmcqGGPaqkaeaacqWGRbWAcqGH9aqpcqaIXaqmaeaacqWGlbWsaiabggHiLdaaaOGaeyypa0tcfa4aaSaaaeaacqaIXaqmaeaacqWGlbWsaaGcdaaeWbqaaiabdohaZnaaDaaaleaacqWGNbWzcqWGRbWAaeaacqaIYaGmaaaabaGaem4AaSMaeyypa0JaeGymaedabaGaem4saSeaniabggHiLdaaaa@6043@

where sgk2
 MathType@MTEF@5@5@+=feaafiart1ev1aaatCvAUfKttLearuWrP9MDH5MBPbIqV92AaeXatLxBI9gBaebbnrfifHhDYfgasaacPC6xNi=xH8viVGI8Gi=hEeeu0xXdbba9frFj0xb9qqpG0dXdb9aspeI8k8fiI+fsY=rqGqVepae9pg0db9vqaiVgFr0xfr=xfr=xc9adbaqaaeGacaGaaiaabeqaaeqabiWaaaGcbaGaem4Cam3aa0baaSqaaiabdEgaNjabdUgaRbqaaiabikdaYaaaaaa@3117@ is the sample variance for the *n*_*k*_observations in each sample set *k*. In this study, *n*_*k *_= 3 for all *k*, so the pooled variance is simply the arithmetic average of all residual variances. *K *= 11 for both the tissue and mixture experiment. See the "Two Groups: Affymetrix" section of the limma user's guide for a description of a comparisons of 2 sets of samples, as used for all comparisons in the detection rate and pAUC analyses. ROC curves were calculated using the ROCR package.

## Authors' contributions

MDR performed the data analysis and drafted the manuscript. TPS initiated the project and offered statistical guidance. Both authors have read and approve the final manuscript.

## Supplementary Material

Additional file 1 Supplementary Materials. This file contains additional figures, tables and comments relevant to the comparison presented in the paper. Click here for file
