# Supplementary material for: A comparison of Affymetrix gene expression arrays
Source: BMC Bioinformatics. 2007 Nov 15;8:449. doi: 10.1186/1471-2105-8-449 (PMC2216046; doi:10.1186/1471-2105-8-449)
Supplement: Additional file 1 — Supplementary Materials. This file contains additional figures, tables and comments relevant to the comparison presented in the paper. [file 1471-2105-8-449-S1.pdf]

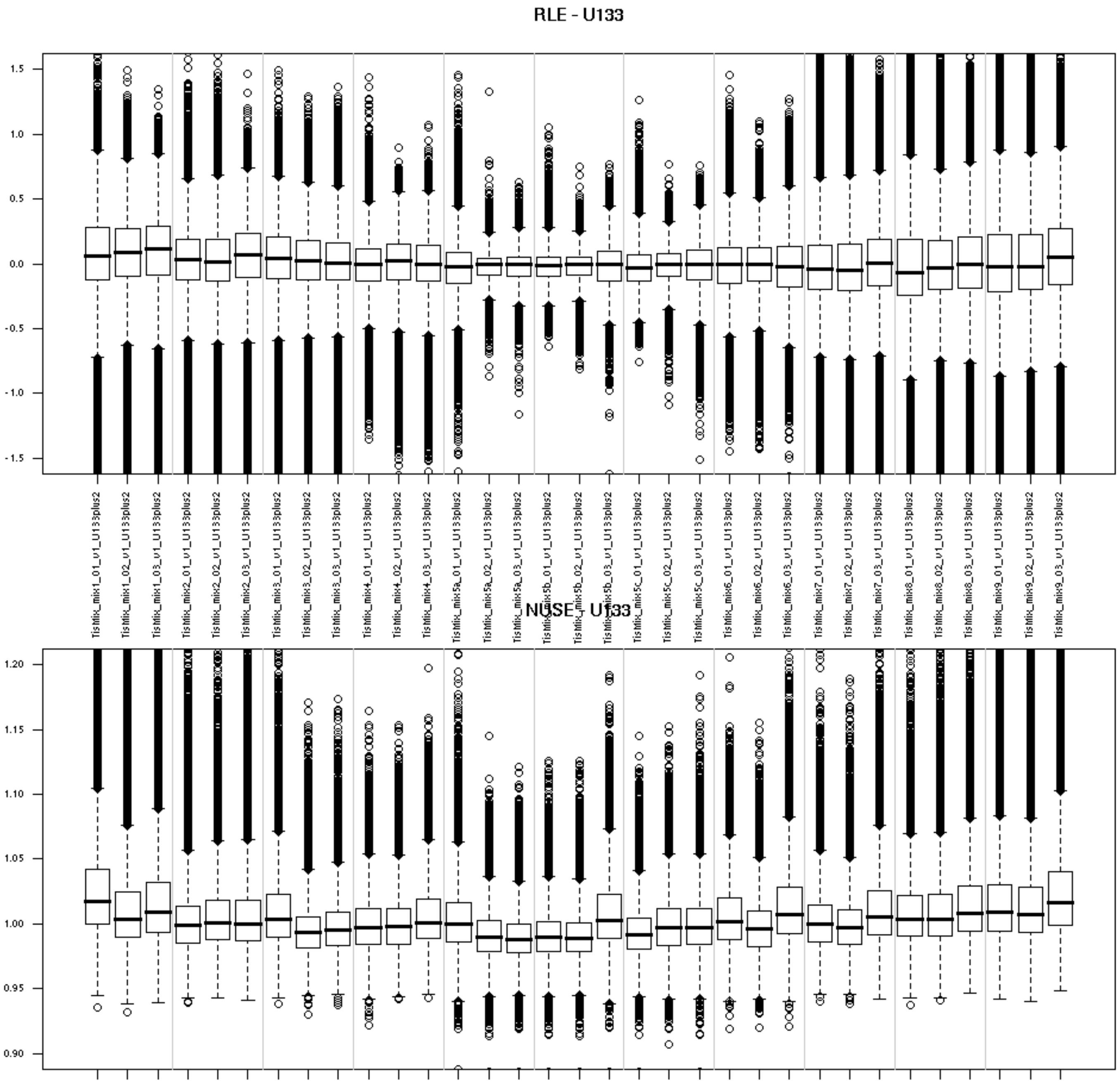

The top panel gives relative log expression (RLE) plots and the bottom panel gives normalized unscaled standard errors (NUSE) plot for the mixture samples on the **U133** array. See Methods for more details. This fanning effect we observe in the RLE plots is expected. For each gene, a median expression value is calculated and RLE is the distribution of log ratio of each sample's expression values compared to median values. One might expect the medians to be quite close to the 50-50 mixture. As the difference from 50-50 increases, we would expect to see more differential expression.

Supplementary materials - page 1

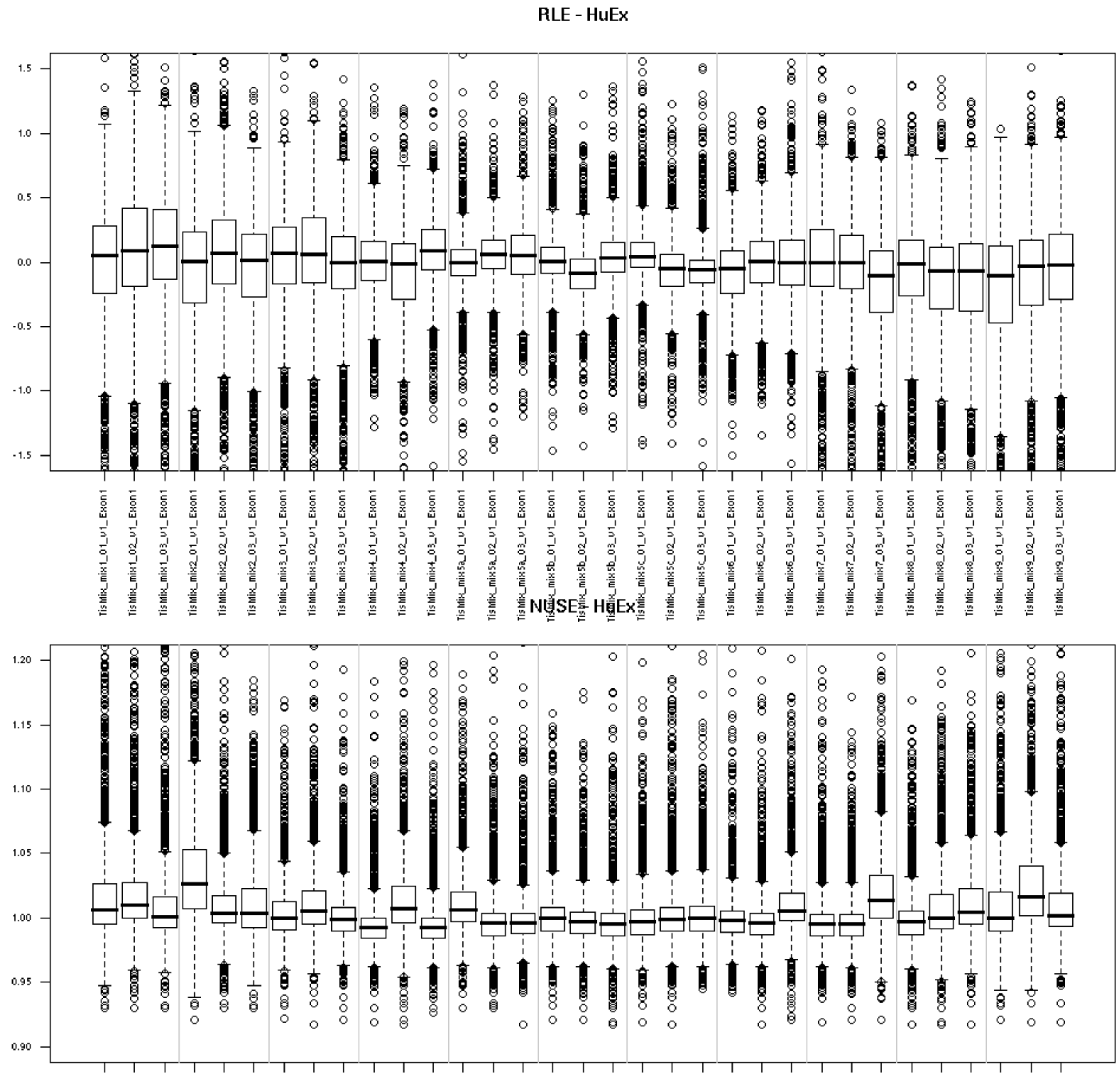

The top panel gives relative log expression (RLE) plots and the bottom panel gives normalized unscaled standard errors (NUSE) plot for the mixture samples on the **HuEx** array.

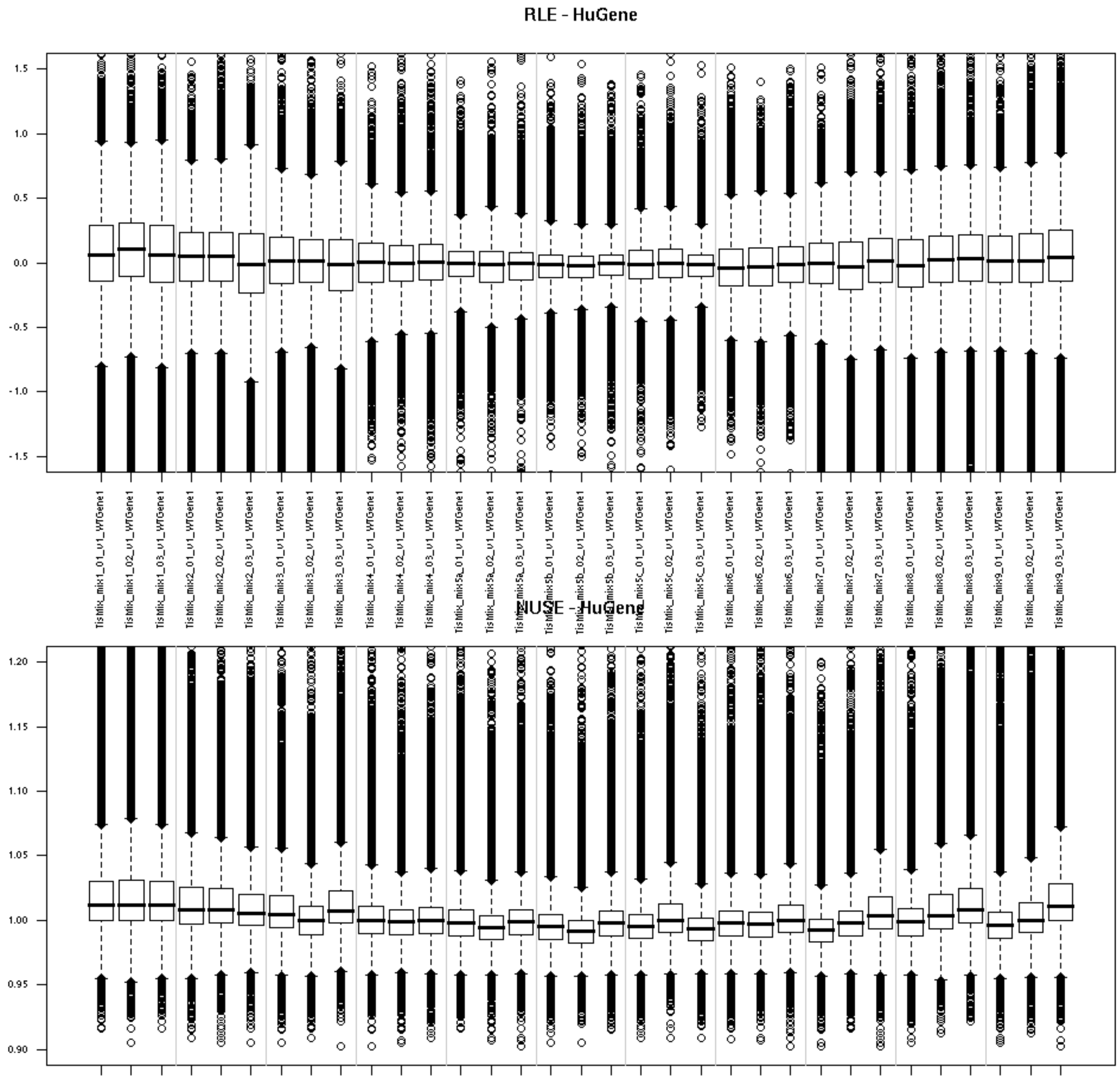

The top panel gives relative log expression (RLE) plots and the bottom panel gives normalized unscaled standard errors (NUSE) plot for the mixture samples on the **HuGene** array.

Mixture	Mix1	Mix2	Mix3	Mix4	Mix 5a	Mix 5b	Mix 5c	Mix6	Mix7	Mix8	Mix9
heart --->	1	0.95	0.9	0.75	0.5	0.5	0.5	0.25	0.1	0.05	0
brain --->	0	0.05	0.1	0.25	0.5	0.5	0.5	0.75	0.9	0.95	1
U133	X		?			X		X			
HuEx		X		X	X			X	X	?	X
HuGene			?						X	X	X

In groups of 3 (technical replicate) samples, three different subjectively chosen levels of hybrizidation quality were assigned: bad (denoted by X), questionable (denoted by ?) or OK. In Supplementary Figures 9-12, the dots are plotted in three sizes: comparisons involving two good chips are largest, comparisons involving at least one questionable chip are medium-sized and comparisons involving at least one bad chip are plotted smallest.

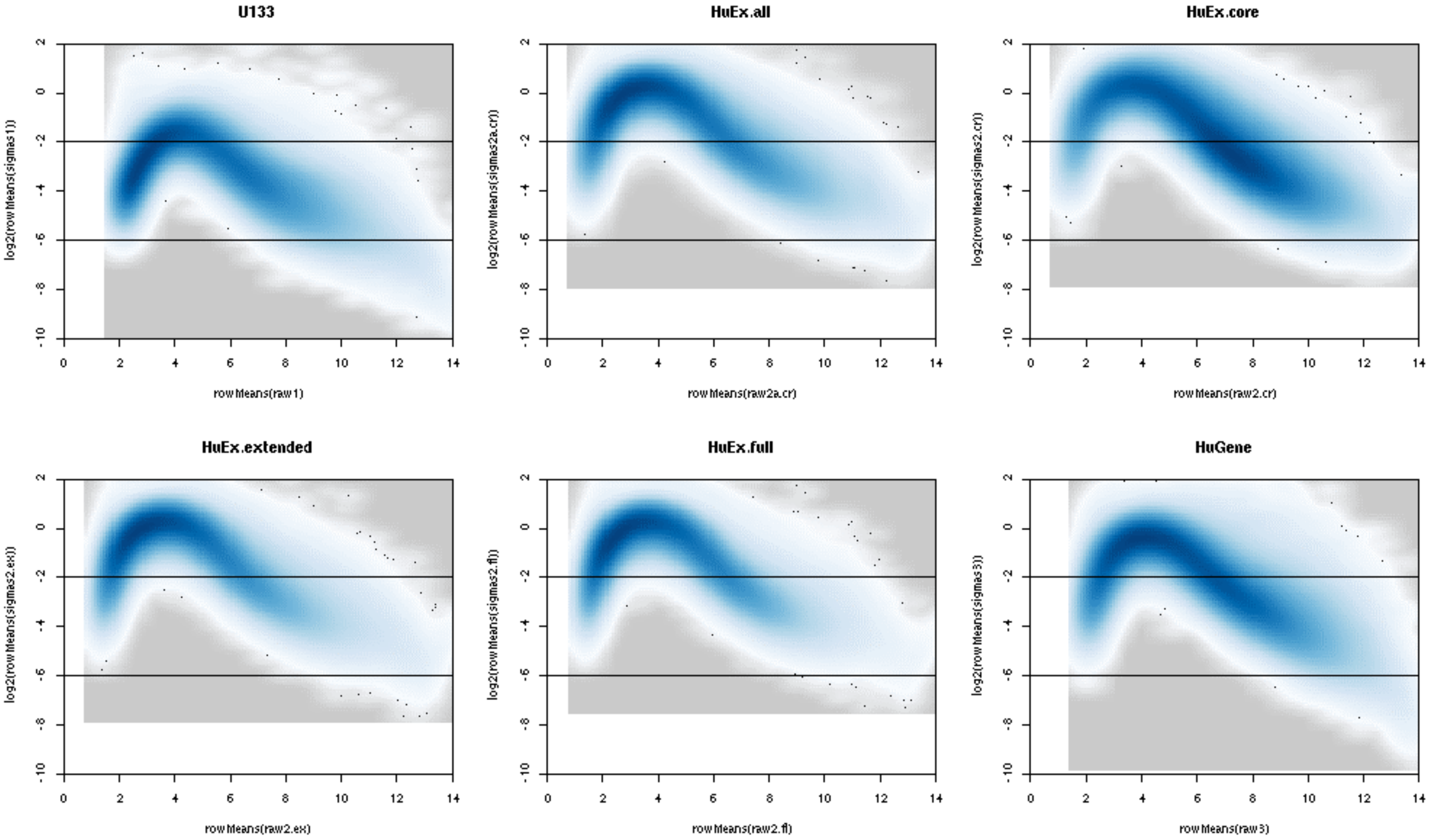

Plots of the probe-level mean-variance relationship for different sets of probes over the mixture dataset: U133 probes, all HuEx probes as well as the core, extended and full subsets and the HuGene probes. X-axis gives the mean over all 33 observations. Y-axis gives the pooled residual variance.

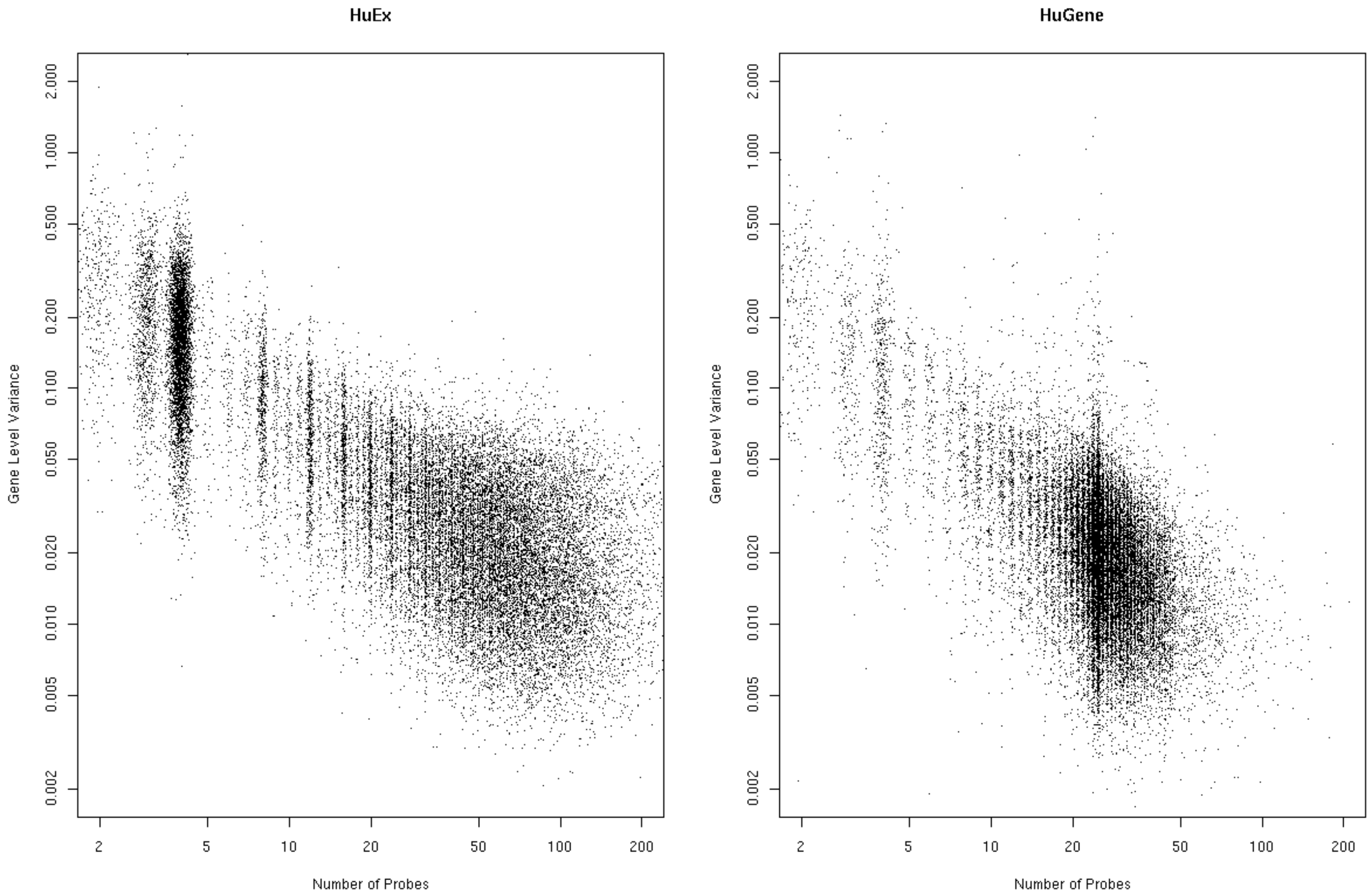

Supplementary materials - page 6

Scatter plot of the gene-level variance to the number of probes. Jitter is added to the X-axis.

Histograms on the number of probes per gene for Ensembl-based probesets.

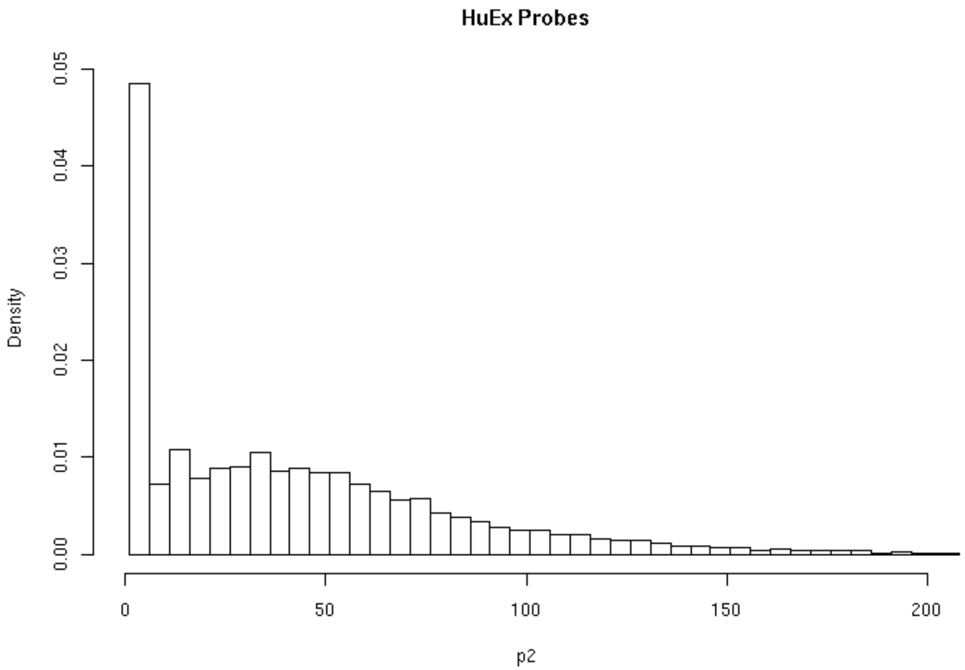

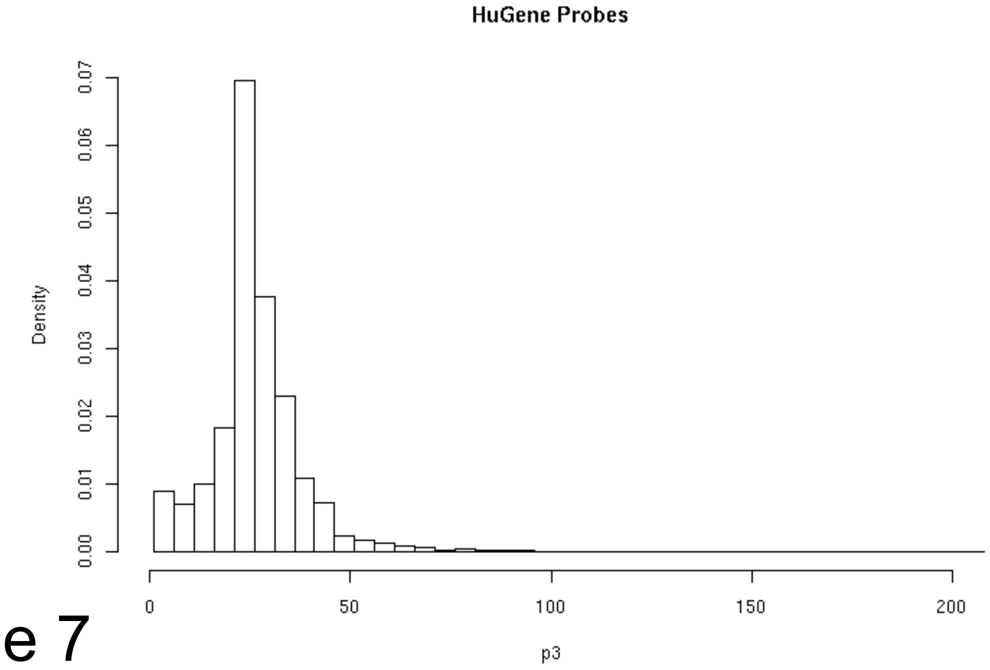

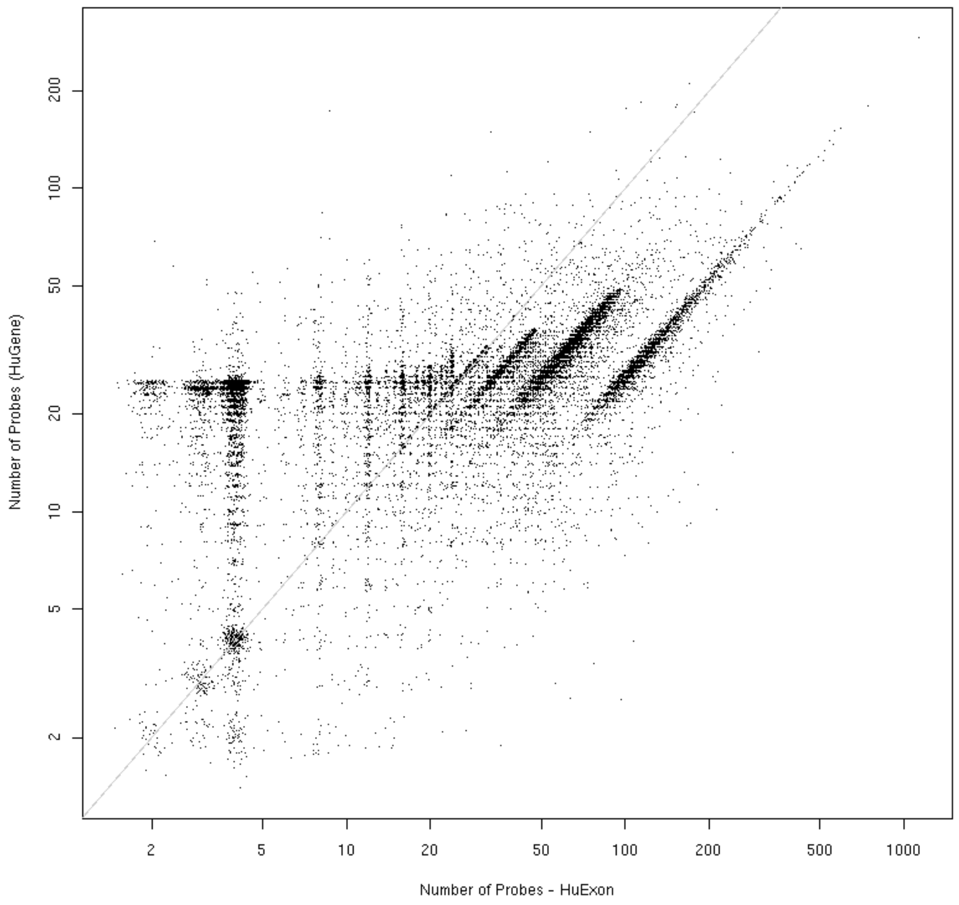

Scattor plot of the number of probes for each Ensembl-based probesets on the HuEx and HuGene platforms. Jitter is added in both axes so that the density of points which overlap can be observed. There are a large number (>4,000) of genes which have few exons (few HuEx probes) but >10 HuGene probes.

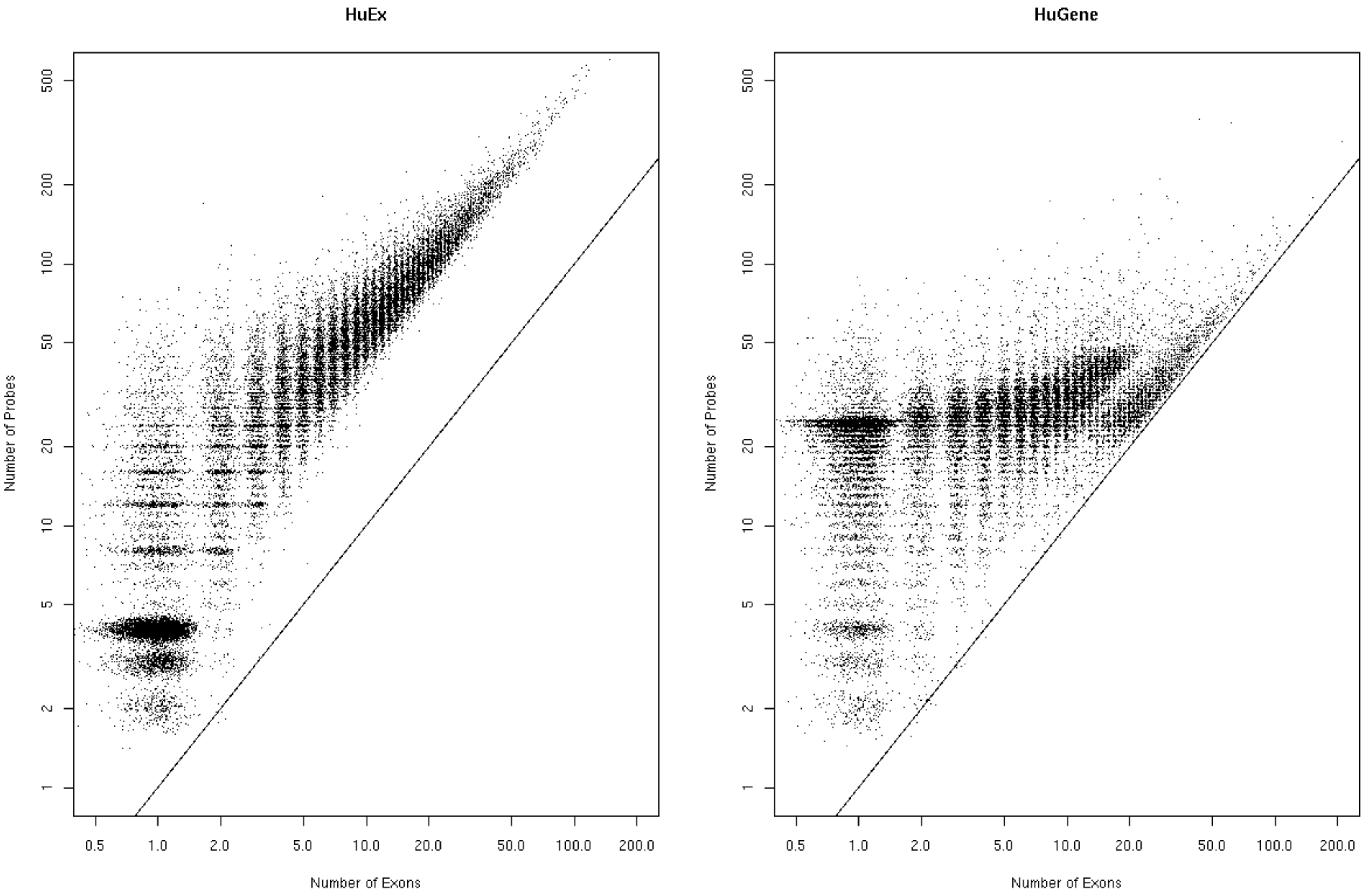

Scattor plot of the number of probes versus the number of exons for each gene for the HuEx and HuGene platforms. Jitter is added in both axes so that the density of points which overlap can be observed.

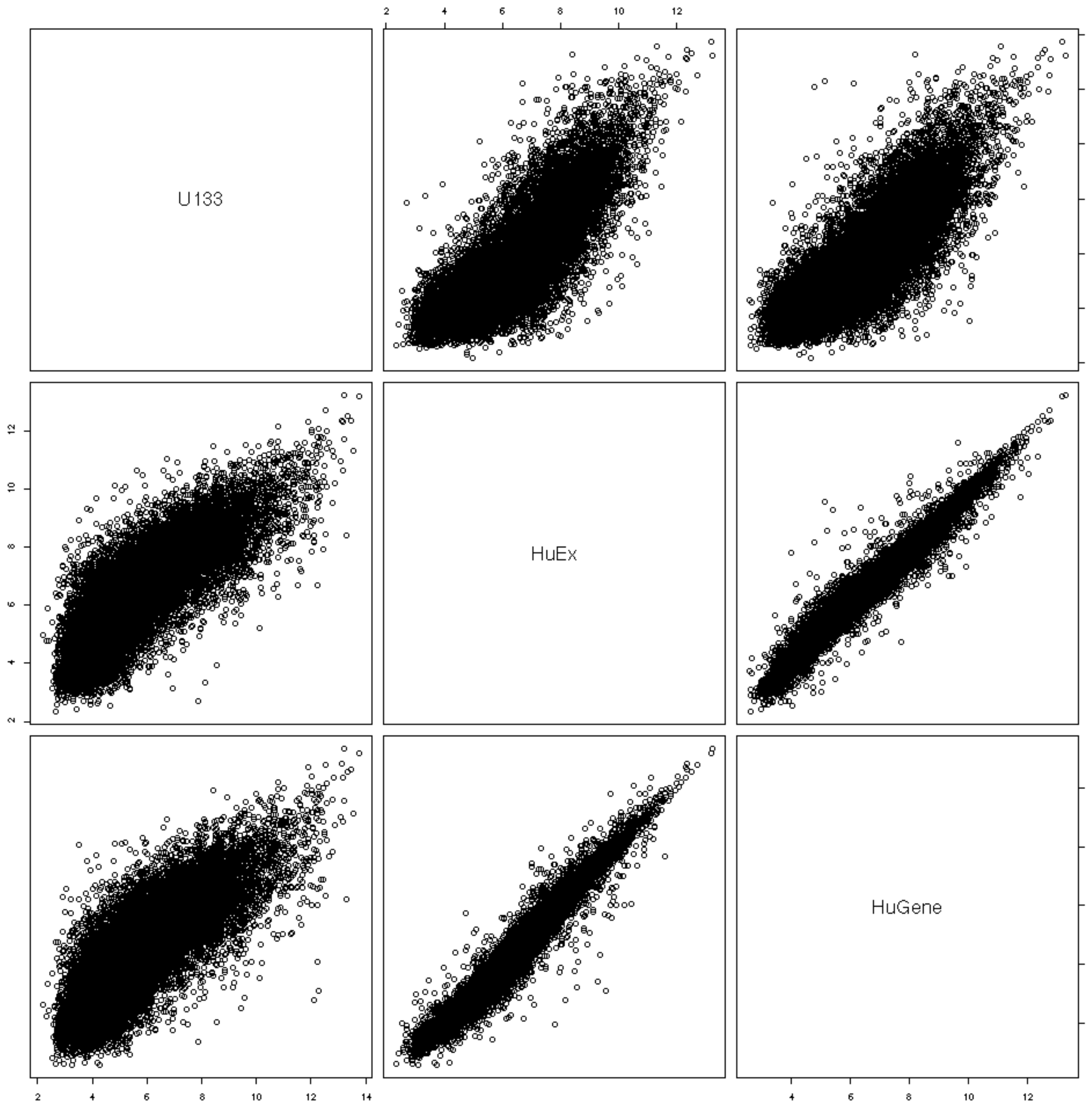

Scatter plot of the average gene-level expression summaries for each of the 3 platforms for Mixture 1.

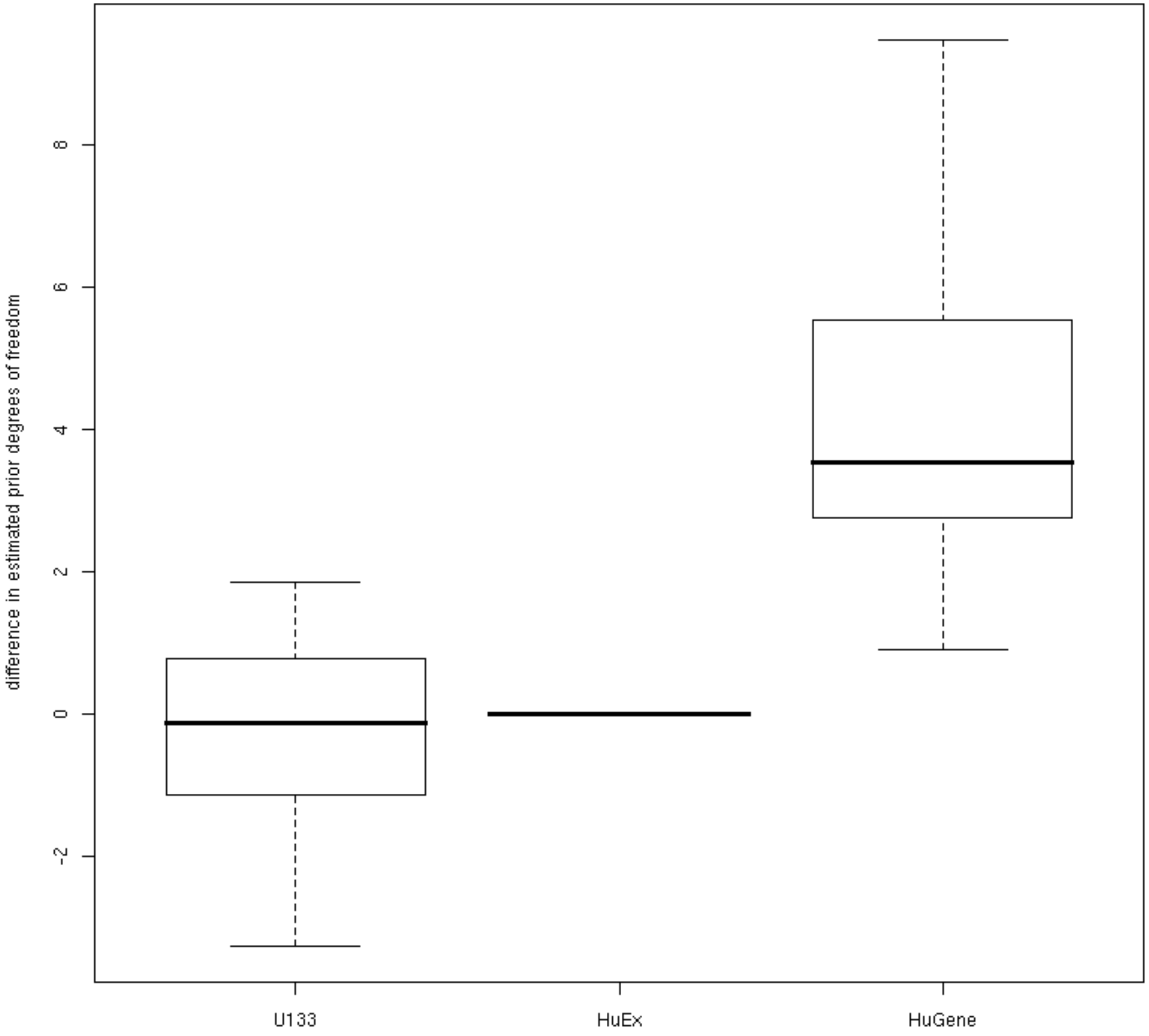

Boxplots of the difference in the number of estimated degrees of freedom (to HuEx) over all 36 pairwise comparisons not including the pure heart and pure brain samples. A higher estimated prior degrees of freedom, which is a reflection of the uniformity of the sample variances, is always observed for HuGene comparisons.

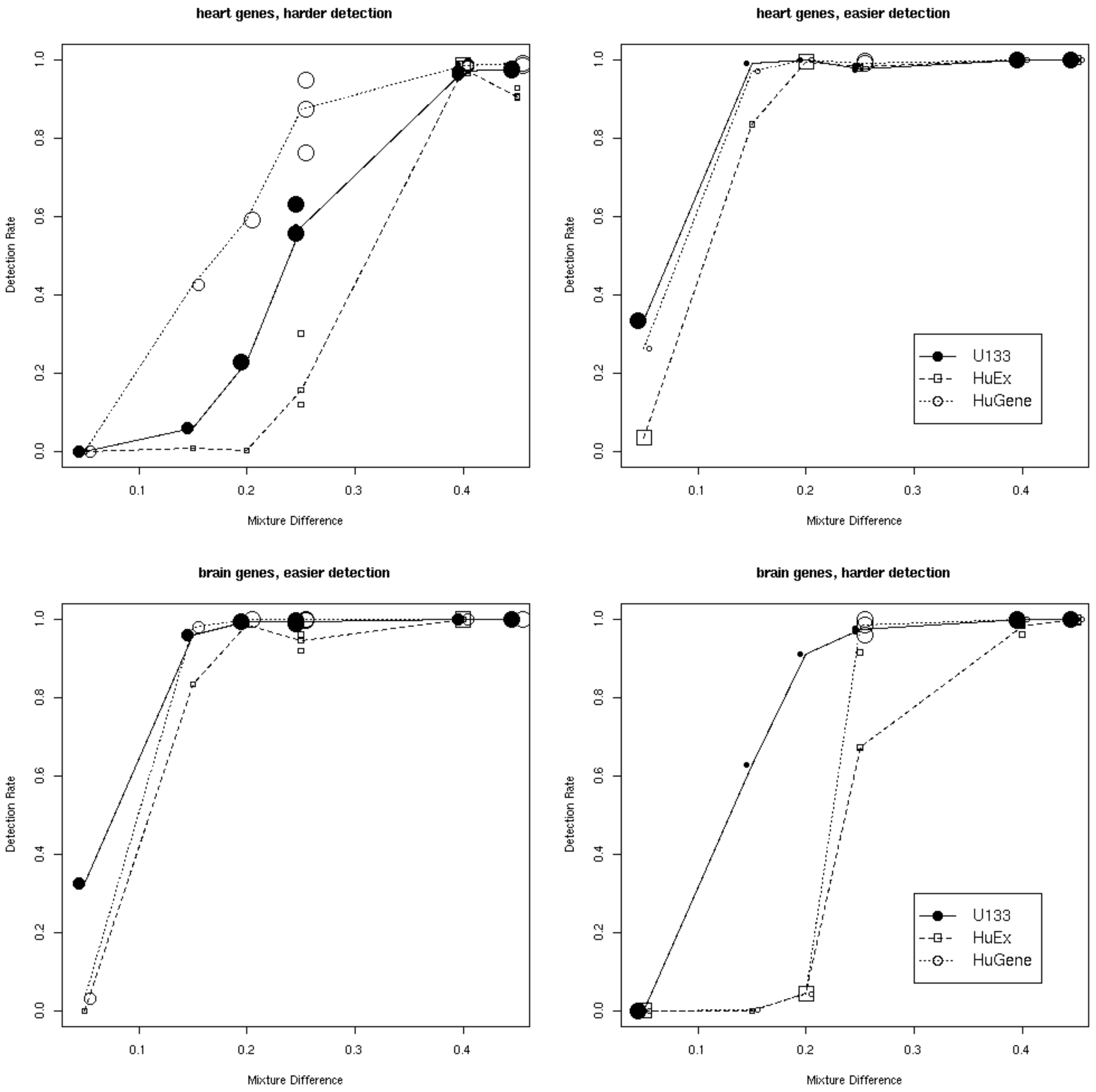

Detection Rates (proportion of previously selected ``true'' genes for each platform that are below the set FDR cutoff) stratified by brain and heart and ``easy'' and ``hard'' detection problems. X-axis is the mixture difference of the comparison. Y-axis gives the detection rate. The top 1000 genes are selected from the pure mixtures. Smaller sized dots signify at least one “questionable” (medium-sized dots) or “bad” (small-sized dots) sets of hybridizations, according to Supplementary Table 1.

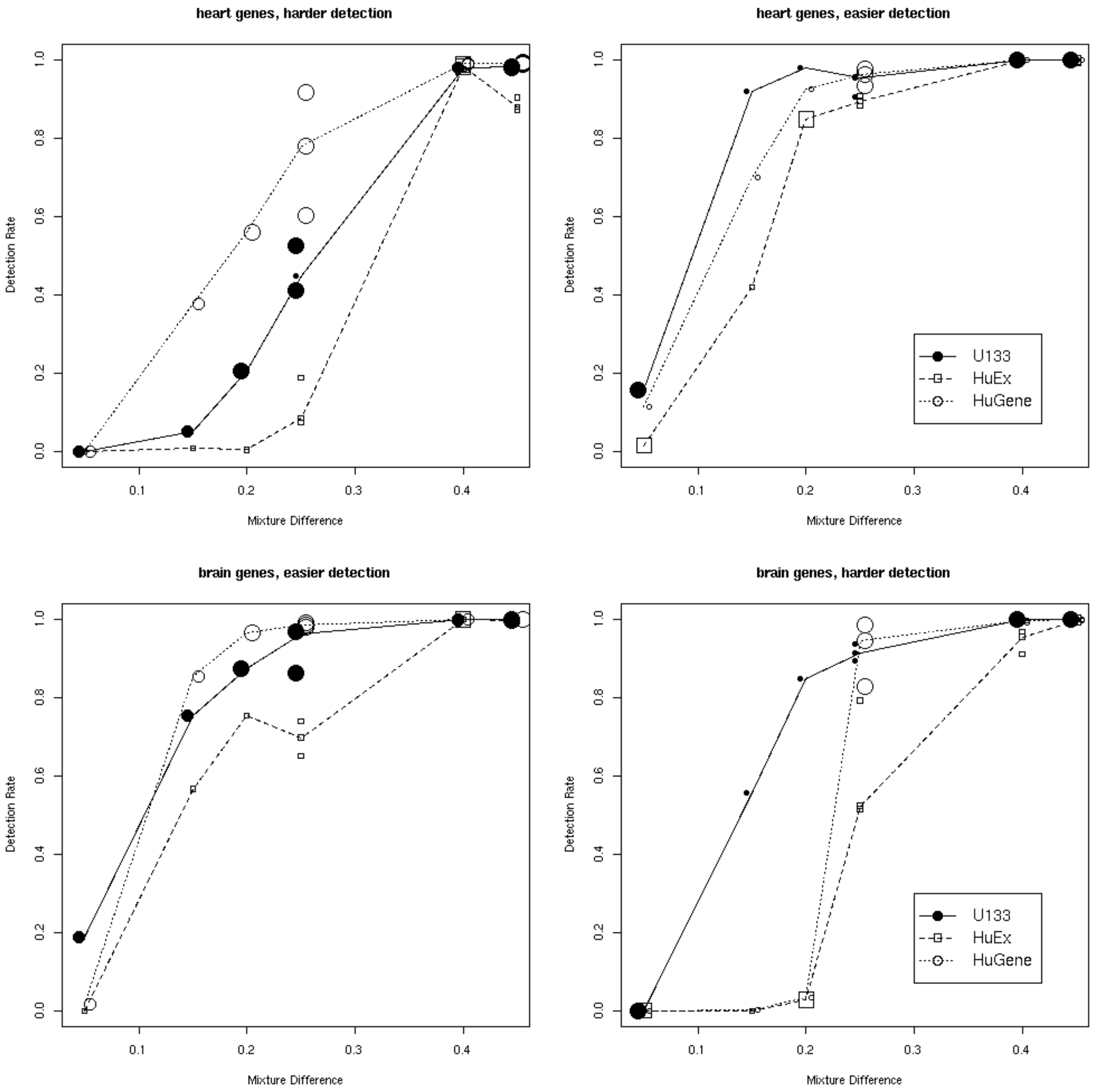

Detection Rates (proportion of previously selected ``true'' genes for each platform that are below the set FDR cutoff) stratified by brain and heart and ``easy'' and ``hard'' detection problems. X-axis is the mixture difference of the comparison. Y-axis gives the detection rate. The top 2000 genes are selected from the pure mixtures. Smaller sized dots signify at least one “questionable” (medium-sized dots) or “bad” (small-sized dots) sets of hybridizations, according to Supplementary Table 1.

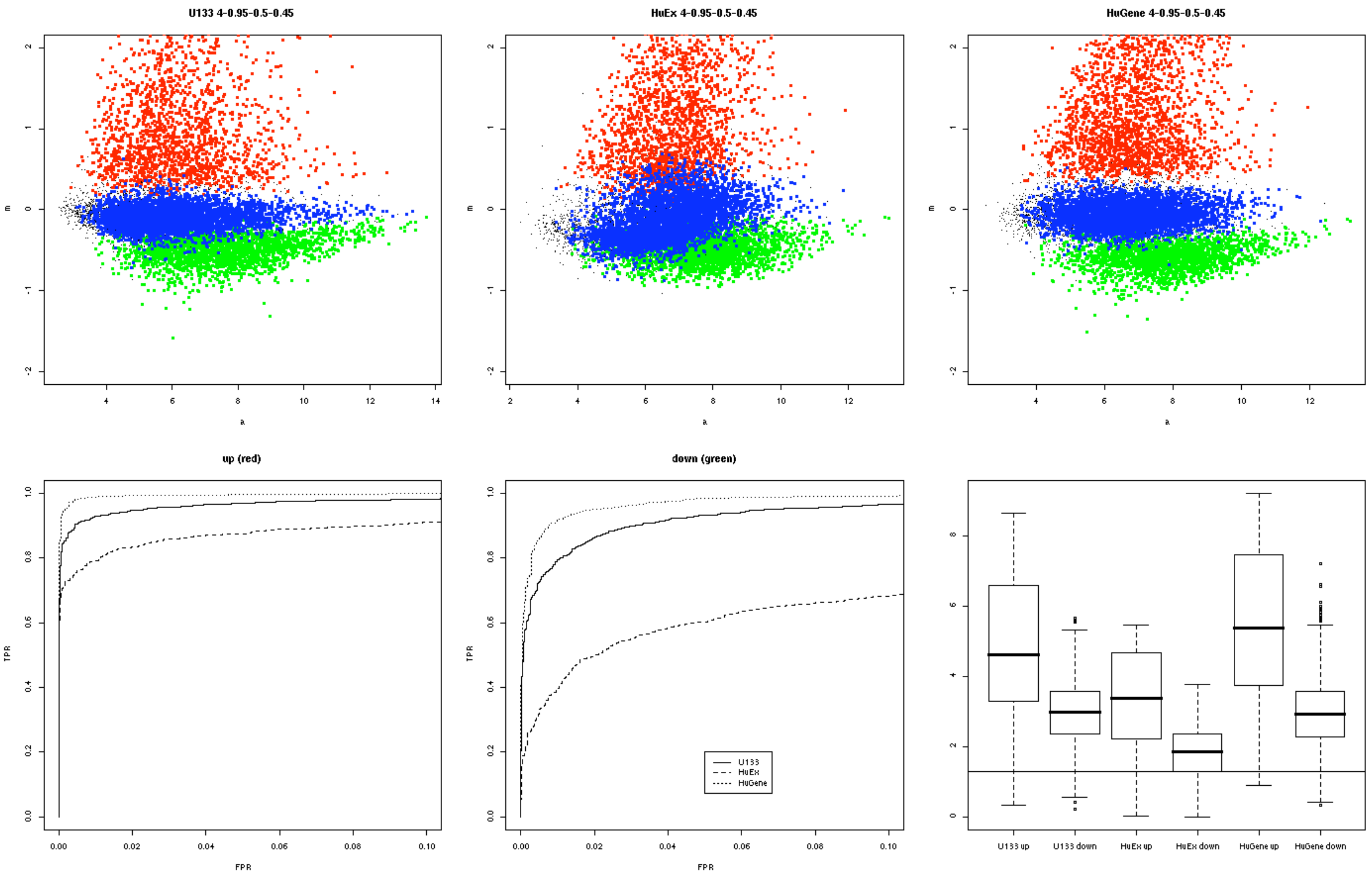

Top 3 panels show MA plots for the 3 platform for a single comparison. Left two panels in the bottom show the ROC curves for separating the truly DE up-regulated genes (in red) and the true down-regulated genes (green) from the non-DE genes. The bottom right panel shows the distribution of the -log10(Adjusted P-values) for the truly DE genes, with the 5% FDR cutoff marked on, thus illustrating the detection rates.

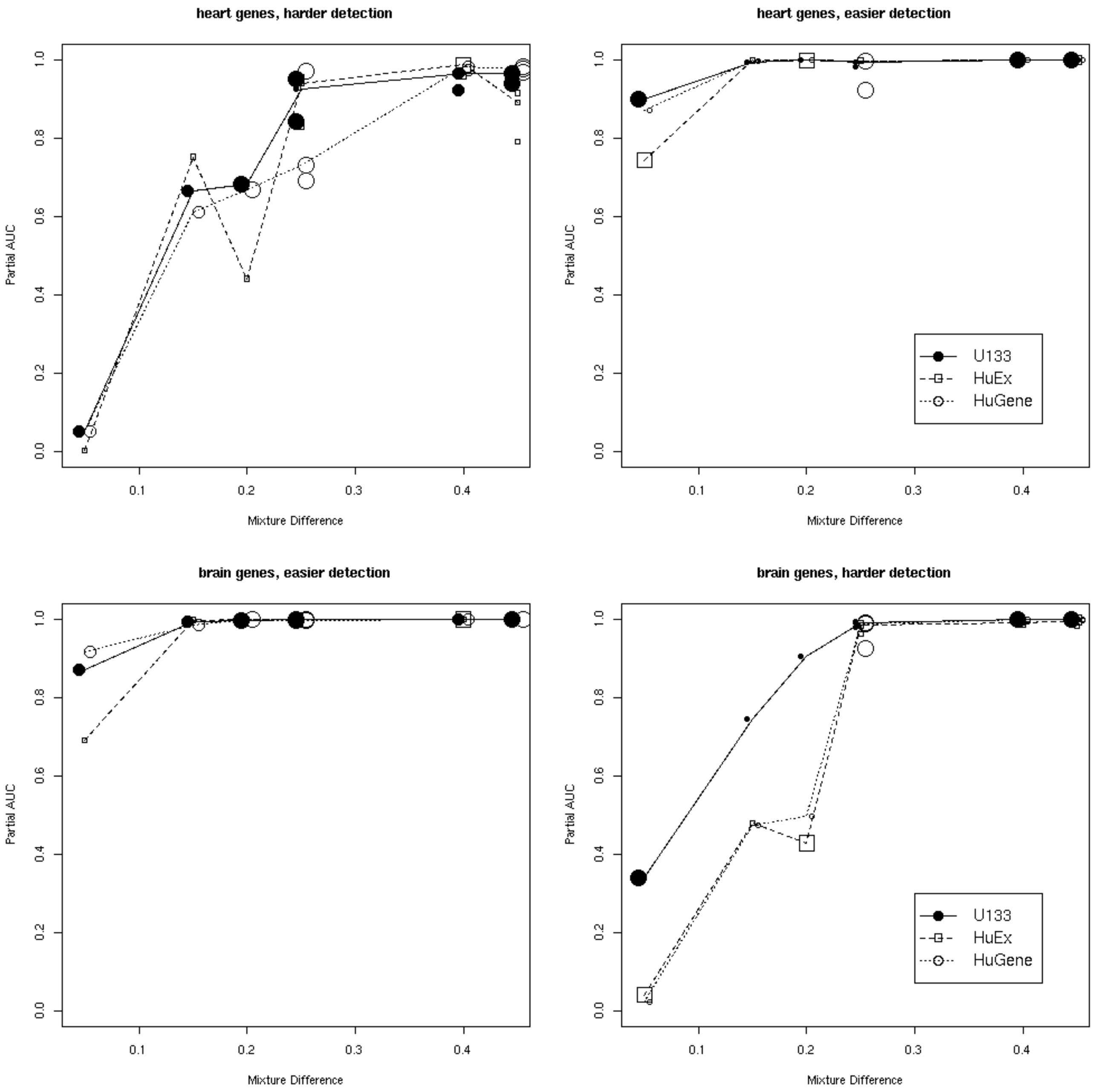

Partial AUCs (area under the ROC curve up to FP=10\%, normalized to have a maximum possible value of 1) stratified by brain and heart and ``easy'' and ``hard'' detection problems. X-axis is the mixture difference of the comparison. Y-axis gives the pAUC. The top 1000 genes are selected from the pure mixtures. Smaller sized dots signify at least one "questionable" (medium-sized dots) or "bad" (small-sized dots) sets of hybridizations, according to Supplementary Table 1.

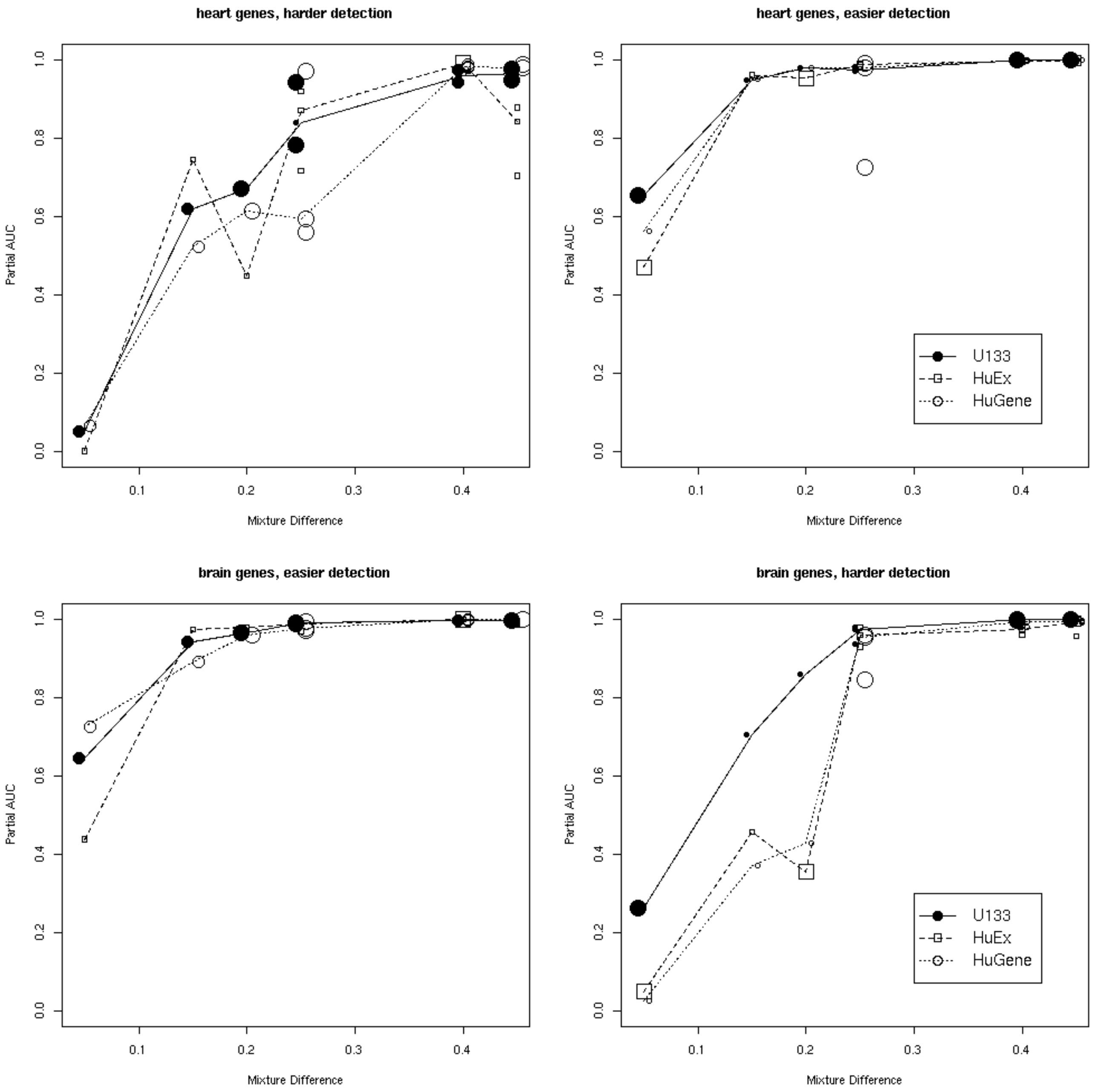

Partial AUCs (area under the ROC curve up to FP=10\%, normalized to have a maximum possible value of 1) stratified by brain and heart and ``easy'' and ``hard'' detection problems. X-axis is the mixture difference of the comparison. Y-axis gives the pAUC. The top 2000 genes are selected from the pure mixtures. Smaller sized dots signify at least one “questionable” (medium-sized dots) or “bad” (small-sized dots) sets of hybridizations, according to Supplementary Table 1.
